# ﻿Review of the European *Eumenes* Latreille (Hymenoptera, Vespidae) using morphology and DNA barcodes, with an illustrated key to species

**DOI:** 10.3897/zookeys.1143.94951

**Published:** 2023-01-31

**Authors:** Cornelis van Achterberg, John T. Smit, Toshko Ljubomirov

**Affiliations:** 1 Naturalis Biodiversity Center, P.O. 9517, 2300 RA Leiden, Netherlands Naturalis Biodiversity Center Leiden Netherlands; 2 European Invertebrate Survey – Netherlands, P.O. 9517, 2300 RA Leiden, Netherlands European Invertebrate Survey Leiden Netherlands; 3 Institute of Biodiversity and Ecosystem Research, Bulgarian Academy of Sciences, Tzar Osvoboditel Boulevard 1, 1000 Sofia, Bulgaria Institute of Biodiversity and Ecosystem Research, Bulgarian Academy of Sciences Sofia Bulgaria

**Keywords:** Biology, *COI* barcode, Eumeninae, new synonymy, potter wasp, systematics, taxonomy, variation

## Abstract

The European species of the potter wasp genus *Eumenes* Latreille, 1802 (Vespidae, Eumeninae) are illustrated and a new illustrated key to the 13 recognised species is presented. *Eumenesmediterraneusaemilianus* Guiglia, 1951 is synonymised with *E.papillarius* (Christ, 1791) (**syn. nov.**), *E.obscurus* André, 1884 and *E.andrei* Dalla Torre, 1894 with *E.pedunculatus* (Panzer, 1799) (**syn. nov.**) and *E.crimensis* Blüthgen, 1938 with *E.sareptanus* André, 1884 (**syn. nov.**).

## ﻿Introduction

The potter wasp genus *Eumenes* Latreille, 1802 (Vespidae, Eumeninae) is distributed nearly worldwide and one of the most common genera of Eumeninae foraging on small flowers with easily accessible nectar in southern Europe. The genus currently includes ca. 106 described species (plus 46 subspecies) divided into two subgenera: subgenus Eumenes Latreille (including all European species) and the small Neotropical subgenus Zeteumenoides Giordani Soika, 1972 with very slender first metasomal tergite (Grandinete et al. 2018). Systematics of the West Palaearctic *Eumenes* species has been confused for a long time; it is considered a “difficult” genus (Gusenleitner 1972) and often a large part of *Eumenes* in collections remains unidentified or is incorrectly named despite the keys by Gusenleitner (1972, 1999) which are a major step forwards compared with the older keys. In most existing keys the considerable variation of nearly all characters used for identification is not or insufficient accounted for, including the influence of the distinct sexual dimorphism. In such cases the best solution is to use a holistic approach; in practise, use combinations of characters and expect at least some deviation for every character. Most essential is to have a good reference collection and excellent illustrations of each sex of the supposed species available. The used reference specimens for this paper were identified by van der Vecht, Blüthgen, Giordani Soika, and Gusenleitner and, in addition, photographs of primary types were examined. In the key below all characters are illustrated per couplet to facilitate identifications. High quality illustrations in the key are essential, otherwise it will be hard to appreciate the subtle differences which may be more or less obscured by variation. The identification is much facilitated when both sexes are available from the same population and if the head is prepared free from the propleuron (i.e., pointing more or less forward). A molecular approach is very helpful (in our case COI sequences as used for barcoding) to check supposed species limits (e.g., Schmid-Egger and Schmidt 2021).

The setosity of body parts is used extensively in existing literature. However, this is a variable and, therefore, rather problematic character, augmented by wear of the setae in aged specimens and concealed setae in wet and/or dirty specimens. In one species (*E.pomiformis*) the regularity of the setosity on the propleuron remains essential for separating it from the very similar *E.subpomiformis*. For all other species additional, though also variable, characters are presented. In most cases a reliable identification will be possible when several complete specimens of each sex of a population are available.

Females build nests consisting of one or several jug like mud cells (Fig. 2), which are sometimes covered with an additional mud layer. The cells are attached to plant stems or stones (Fig. 2b), but *E.papillarius* females arrange the mud cells in small groups under roof tiles or in other more or less protected places. Rarely pre-existing cavities are used for constructing the mud cells. The mud cells are provisioned with small caterpillars, usually belonging to Geometridae or various microlepidopterous families (Fateryga 2017).

## ﻿Materials and methods

Identified material was used from the following collections: Naturalis Biodiversity Center (Leiden; **RMNH**), Biologiezentrum of the Oberösterreichisches Landes-Kultur GmbH (Linz; **BZL**), J. Smit collection (Duiven; **SCD**), Institute of Biodiversity and Ecosystem Research (Sofia; **IBER**) and National Museum of Natural History (Sofia; **NMNHS**). Additional specimens were collected in Bulgaria, Greece, Italy, Netherlands, and Turkey by the first author since 1998 and deposited in RMNH. Two COI sequences were used of specimens deposited in the Finnish Museum of Natural History (Helsinki; **FMNH**). Photographs were taken with a Canon 5Ds 50.6-megapixel camera combined with a Canon MP-E 65 mm f/2.8 1–5× Macro lens, Laowa Macro Twin flash KX-800 and an electronic WeMacro Z-stepper rail. The photographs were stacked with Helicon Focus 7 software. An asterisk indicates a new record for the country. Additional photographs of types were received from Museo Civico di Storia Naturale “G. Doria”, Genova, Italy (**MSNG**; Roberto Poggi), Natural History Museum of Denmark, Copenhagen, Denmark (**NHMD**; Sree Gayathree Selvantharan), Eidgenössische Technische Hochschule, Zürich, Switzerland (**ETHZ**; Michael Greeff) and Zoologische Staatssammlung, München, Germany (**ZSM**; Stephan and Olga Schmidt).

**Figure 1.**
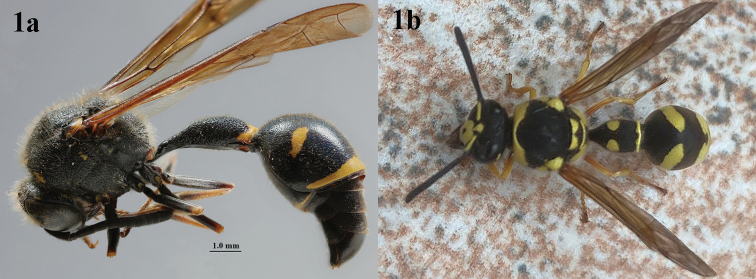
Showing dark (**1a**) and pale (**1b**) type of species **1a***Eumenescoronatus* (Panzer), female, Finland, habitus lateral **1b***E.pomiformis* (Fabricius), female, Bulgaria, habitus dorsal. Photographs: C. van Achterberg.

DNA extraction was conducted on single legs, using the NucleoMag 96 Tissue kit by Macherey-Nagel on a Thermo Scientific KingFisher Flex magnetic bead extraction robot, with a final elution volume of 150 µl. The standard COI barcoding fragment (Hebert et al. 2003) was amplified using a cocktail of primers LCO1490 and HCO2198 (Folmer et al. 1994), and LepF1 and LepR1 (Hebert et al. 2004). PCR reactions contained 18.75 µl mQ, 2.5 µl 10× PCR buffer CL, 1.0 µl 10 mM of each primer, 0.5 µl 2.5 mM dNTPs and 0.25 µl 5U Qiagen Taq, with 1.0 µl of template DNA. PCR was performed using an initial denaturation step of 180 s at 94 °C, followed by 40 cycles of 15 s at 94 °C, 30 s at 50 °C and 40 s at 72 °C, and finishing with an extension of 300 s at 72 °C and pause at 12 °C. Bidirectional sequencing was performed at BaseClear (http://www.baseclear.com/). Sequences were edited manually with Sequencher 4.10.1 (Gene Codes Corporation). For all barcoded specimens, sequences and collection data were uploaded to the Barcode of Life Database (BOLD; http://www.boldsystems.org/). The voucher specimens are deposited in RMNH and the collection of J. Smit. BOLD accession codes are provided for the specimens that produced DNA barcodes in Table 1.

**Figure 2.**
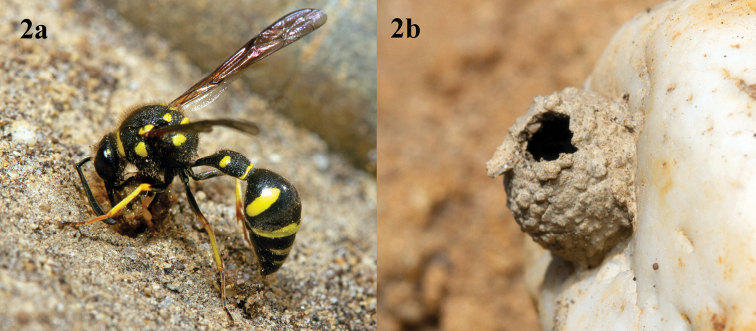
**2a***Eumenescoarctatuscoarctatus* (Linnaeus), female, Netherlands, collecting clay for nest construction **2b** abandoned clay nest. Photographs: J.T. Smit.

**Table 1.** Sampled specimens from RMNH and J. Smit collection, but both dark specimens of *E.coarctatus* from Finland are deposited in FMNH.

Taxon	ID number	BOLD accession number	Country
*Eumenescoronatus*	RMNH.INS.545385	NLHYM109-12	Netherlands
*Eumenes* “*coarctatus*”	RMNH.INS.545482	NLHYM206-12	Netherlands
*Eumenescoarctatus*	RMNH.INS.547000	NLHYM394-12	Netherlands
*Eumenes* “*coarctatus*”	RMNH.INS.547063	NLHYM457-12	Netherlands
*Eumenescoronatus*	RMNH.INS.547399	NLHYM608-12	Netherlands
*Eumenespedunculatus*	RMNH.INS.547306	NLHYM515-12	Netherlands
*Eumenescoarctatus*	RMNH.INS.1092784	NLHYM969-22	Netherlands
*Eumenescoarctatus*	RMNH.INS.1092785	NLHYM970-22	Netherlands
*Eumenespedunculatus*	RMNH.INS.1092786	NLHYM971-22	Netherlands
*Eumeneslunulatus*	RMNH.INS.1092788	NLHYM973-22	Bulgaria
*Eumenesmediterraneus*	RMNH.INS.1092789	NLHYM974-22	Bulgaria
*Eumenespomiformis*	RMNH.INS.1092790	NLHYM975-22	Bulgaria
*Eumenespapillarius*	RMNH.INS.1092791	NLHYM976-22	Bulgaria
*Eumenespapillarius*	RMNH.INS.1092792	NLHYM977-22	Bulgaria
*Eumenescoronatus*	RMNH.INS.1092793	NLHYM978-22	Bulgaria
*Eumenescoronatus*	RMNH.INS.1092794	NLHYM979-22	Bulgaria
*Eumenescoronatus*	RMNH.INS.1092795	NLHYM980-22	Netherlands
*Eumenesmediterraneus*	RMNH.INS.1092796	NLHYM981-22	France
*Eumenesmediterraneus*	RMNH.INS.1092797	NLHYM982-22	Greece
*Eumenesmediterraneus*	RMNH.INS.1092798	NLHYM983-22	Bulgaria
*Eumenessubpomiformis*	RMNH.INS.1092799	NLHYM984-22	Bulgaria
*Eumenespomiformis*	RMNH.INS.1092800	NLHYM985-22	Greece
*Eumenescoarctatus*	MZ626999	ACUFIN803-13	Finland
*Eumenescoarctatus*	MZ627515	ACUFIN804-13	Finland
*Ancistrocerustrifasciatus*	RMNH.INS.1092801	NLHYM034-12	Netherlands

## ﻿Results

### ﻿Molecular data

**Figure 3.**
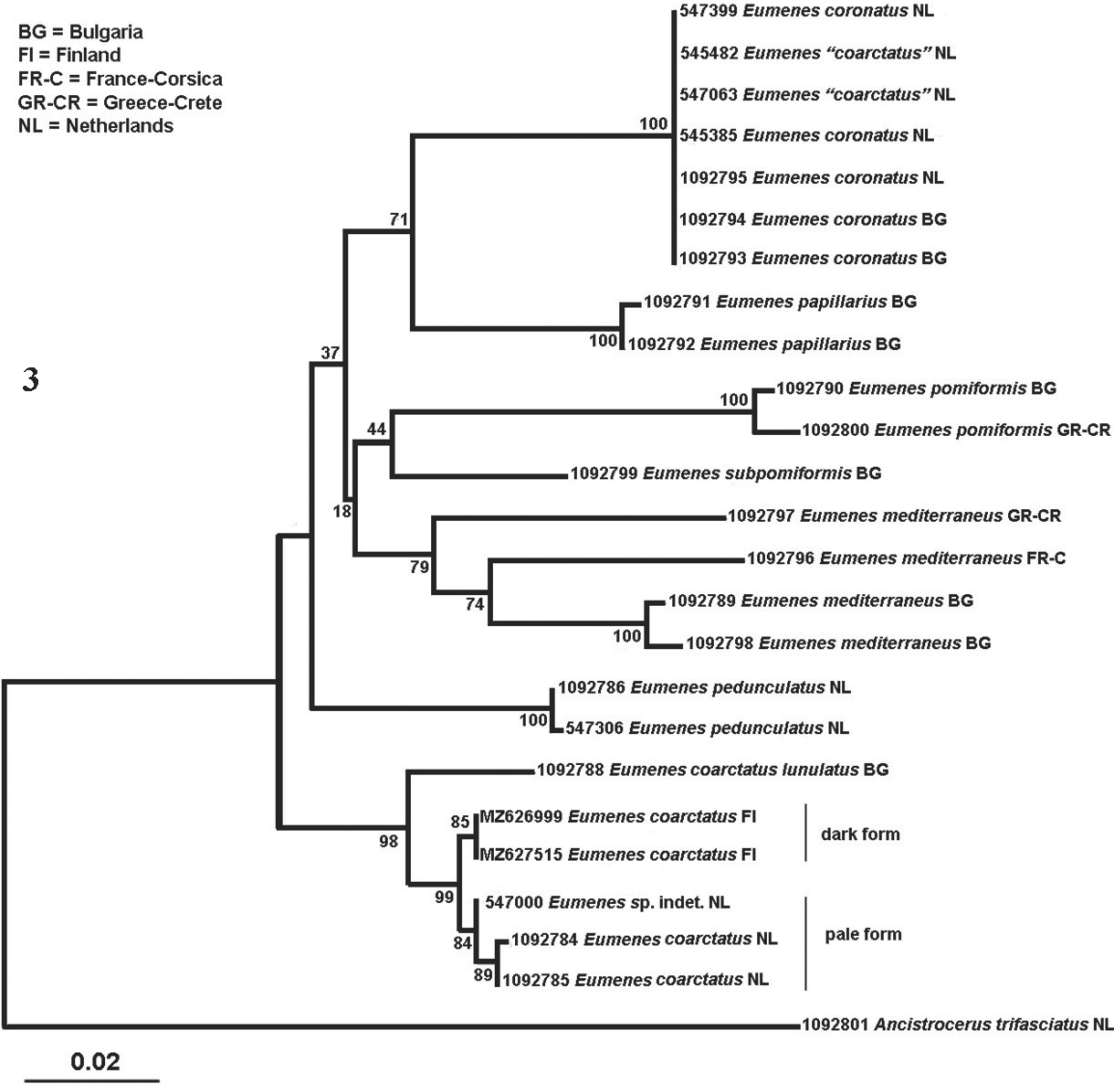
Neighbour-Joining tree for barcoded European *Eumenes* species. Both *E.coarctatus* specimens (sampled 5 y ago for another project and not available for examination) among *E.coronatus* are obviously misidentified and, therefore, in quotation marks. The numbers are RMNH unique identifiers except the Finnish numbers of both dark specimens of *E.c.coarctatus* from Finland.

In the Neighbour-Joining tree (using COI sequences) newly barcoded specimens from Bulgaria, France, Greece, and Netherlands are combined with unpublished older sequences from Finland and Netherlands (Fig. 3). As outgroup we used the related Eumenine *Ancistrocerustrifasciatus* (Müller, 1776). Table 1 contains details of the barcoded specimens, like country of origin, ID number and BOLD accession number.

First of all, it is clear that *E.mediterraneus* is a species complex and the characters used for its recognition seems to be insufficient. The lectotype of *E.mediterraneus* originates from Croatia (Dalmatia) and is most likely the same species as the sampled specimens from Bulgaria. An extensive survey is necessary to find out what the position of the taxa within this complex is.

The position of the *E.coarctatuslunulatus* specimen from Bulgaria in the NJ tree indicates that it is different from the sampled N and NW European specimens of *E.c.coarctatus* (whereas the two colour forms of the latter obviously belong together; Fig. 3). Gusenleitner (1972) treated *E.lunulatus* as a valid species but later (1999) he lowered its rank to subspecies. In general, as noted by Gusenleitner (1972)*Eumenes* species become paler and more coarsely sculptured in southern areas than in boreo-montane areas. Likely this is related to thermoregulation of the body; the paler parts reflect sunlight and the heavily sculptured (and thus heavier sclerotised) areas give more protection then the largely smooth and thinner body parts in boreo-alpine species. *Eumenescoarctatuslunulatus* seems to have a more south-eastern distribution in Europe than the typical *E.coarctatus*. *Eumenesc.lunulatus* is more sculptured and paler, indicating a more south-eastern origin than *E.c.coarctatus*. At the moment it cannot be ruled out that different populations overlap in Central Europe and that we may have to accept that forms with mainly smooth tergites and those with coarse punctures occur in the same taxon as proposed by Neumeyer and Praz (2015) and Neumeyer (2014). Since there is a molecular difference and both taxa or forms seem to exist together in Central Europe, we follow Gusenleitner (1999) and treat *E.lunulatus* as a valid subspecies of *E.coarctatus* until more data will become available.

### ﻿Illustrated key to European species of *Eumenes*

N.B. Species can only be reliably identified by a combination of characters. Setosity may be worn off in aged specimens or hardly visible in dirty specimens; therefore, aged or dirty specimens are easily misidentified. In addition, there is a considerable intraspecific variation.

**Table:** 

1	Females: antenna without terminal hook (a); clypeus often partly or entirely black (b); inner tooth of hind tarsal claw widened and apically more or less truncate (c), but intermediate in *E.coronatus*; [mandibles somewhat larger than in ♂]	**2**
	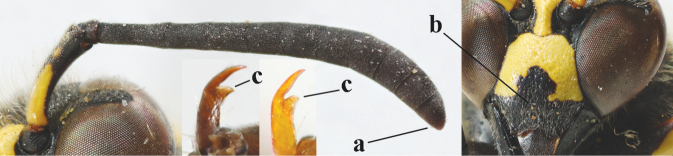	
–	Males: antenna with terminal hook (aa); clypeus nearly always entirely yellow (bb); inner tooth of hind tarsal claw comparatively slender and apically acute (cc)	**16**
	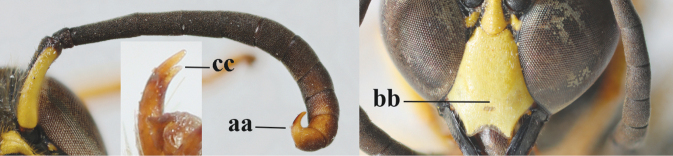	
2	Setae of occiput short to medium-sized (a); second and third antennal segments reddish or brownish ventrally (b), rarely entirely black in SW Europe; [pronotum often broadly yellow laterally]	**3**
	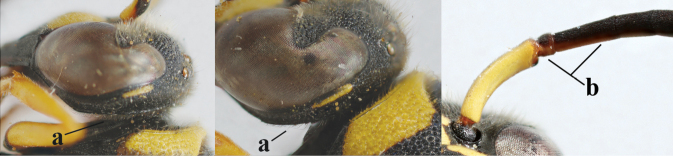	
–	Setae of occiput long (aa; but sometimes worn off); second and third antennal segments black ventrally (bb)	**5**
	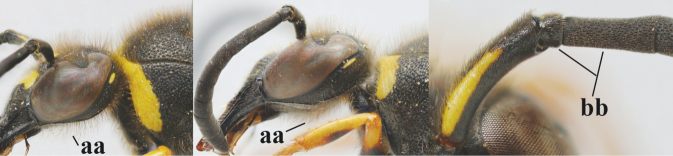	
3	First metasomal segment tricoloured ventrally (a); clypeus entirely yellow (b) or with small medial black patch; parategula yellow (c); [basal half of antenna usually extensively yellowish brown ventrally; pronotum broadly yellow posteriorly]	***E.cyrenaicus* Blüthgen, 1938**
	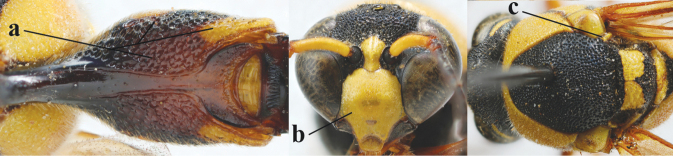	
–	First segment bicoloured ventrally (aa); clypeus partly black (bb); parategula black (cc)	**4**
	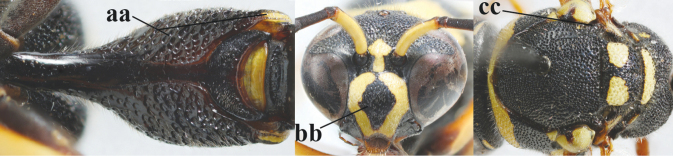	
4	Apical third of clypeus broadly yellow laterally (a); setae of mesoscutum comparatively short anteriorly (b); clypeus less convex compared to face in lateral view (c)	***E.dubius* de Saussure, 1852**
	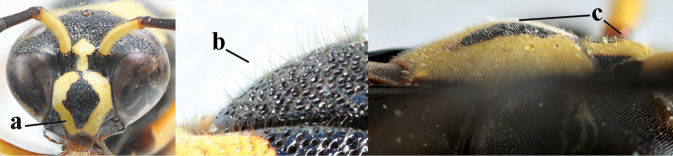	
–	Apical third of clypeus broadly black laterally (aa); setae of mesoscutum comparatively long anteriorly (bb); clypeus more convex compared to face in lateral view (cc)	***E.sareptanus* André, 1884**
	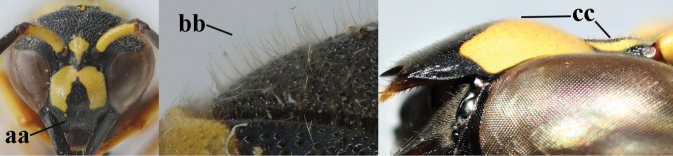	
5	Posterior part of first metasomal tergite comparatively slender in ventral view (a) and weakly convex dorsally in lateral view (b); apical lamella of second tergite often subhyaline or pale yellow (c), **if** dark brown then setae of second tergite medium-sized (d) to long (dd) in lateral view; [hind basitarsus often dark brown basally]	**6**
	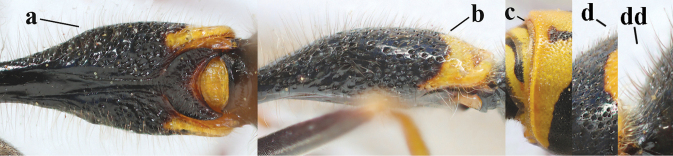	
–	Posterior part of first tergite comparatively robust in ventral view (aa) and more convex dorsally in lateral view (bb); apical lamella of second tergite dark brown or blackish (cc), rarely yellowish; setae of second tergite either long (dd) or inconspicuous in lateral view (ddd)	**10**
	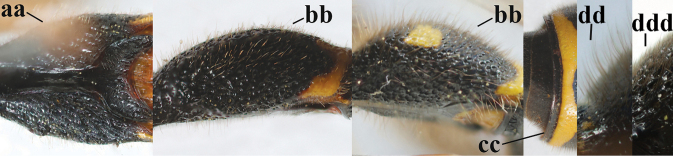	
6	Second metasomal tergite with three large (more or less separated) black spots medially (a); first metasomal segment largely orange or yellow (b); clypeus entirely yellow (c); mesoscutum with medium-sized to large yellow or orange patch laterally (d); [propleuron with short setae]	***E.tripunctatus* (Christ, 1791)**
	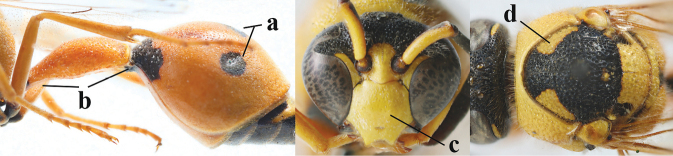	
–	Second metasomal tergite with wide black band medially (aa); first segment mainly black (bb); clypeus at least medially partly black (cc); mesoscutum usually entirely black (dd) or with pair of small to large patches (large specimens of *E.papillarius*)	**7**
	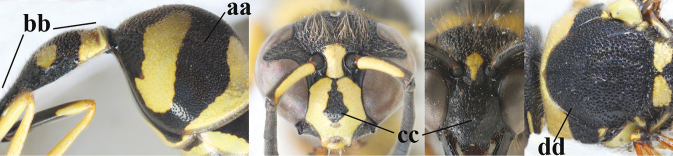	
7	Second metasomal sternite with long setae (a), rarely intermediate; scape entirely black anteriorly (b), rarely with short yellow stripe; inner tooth of hind tarsal claw narrower and rather acute (c); clypeus comparatively deeply emarginate medio-apically (d); [hind tarsus dark brown; dorsal setae of scape often long; second tergite rather remotely punctate and bristly setose]	***E.coronatus* (Panzer, 1799)**
	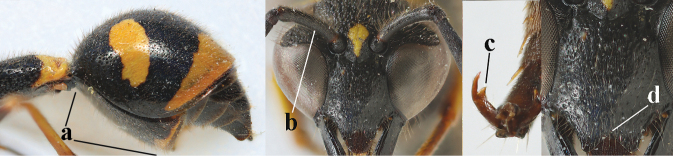	
–	Second sternite with short to medium-sized setae (aa), rarely intermediate (Iberian Peninsula); anteriorly scape (except sometimes apical third or half) yellow (bb); inner tooth of hind tarsal claw wider and distinctly truncate (cc); clypeus less emarginate medio-apically (dd); but comparatively deep in *E.mediterraneus* (ddd)	**8**
	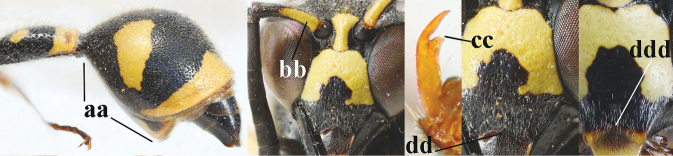	
8	Apical lamella of second tergite subhyaline or pale yellowish (a); ventral corners of clypeus narrower because of deeper medio-apical emargination (b); propodeum usually with medium-sized smooth interspaces (c) and anterior half of median groove distinct (d); second tergite comparatively convex medially and with short setae (e); first tergite slender in dorsal view (f); parategula frequently more or less yellow (g); labrum partly or entirely yellow (h); [hind basitarsus often brown basally]	***E.mediterraneus* Kriechbaumer, 1879**
	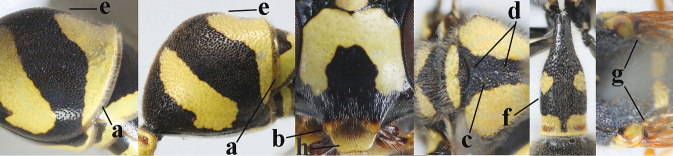	
–	Apical lamella of second tergite brownish or blackish (aa), rarely pale brown or pale yellowish; ventral corners of clypeus wider apically because of shallow medio-apical emargination (bb); propodeum usually without smooth interspaces (cc) and anterior half of median groove largely reduced (dd); second tergite less convex medially and with medium-sized to long setae (ee); first tergite less slender in dorsal view (ff); parategula usually black (gg) or largely so; labrum entirely dark brown or black (hh)	**9**
	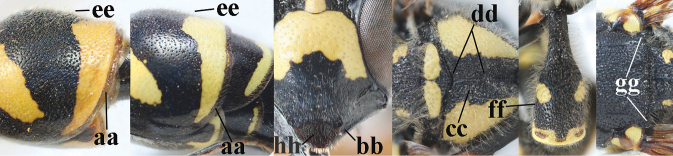	
9	Third tergite and sternite partly yellow posteriorly (a); hind basitarsus more or less darkened basally and outer apex of hind tibia with blackish patch dorsally (b); first metasomal tergite elongate in lateral view (c); first tergite usually less densely punctate subposteriorly (d); second tergite more shiny and usually less densely sculptured laterally (e); [large specimens (fore wing about 10 mm) have mesoscutum frequently with a pair of yellow patches antero-laterally; **if** hind tibia apically and basitarsus basally yellow and second tergite with satin sheen laterally, go to 10]	***E.papillarius* (Christ, 1791)**
	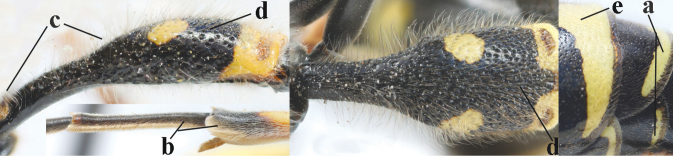	
–	Third tergite and sternite black posteriorly (aa); hind basitarsus and outer apex of hind tibia entirely yellow (bb); first tergite less elongate in lateral view (cc); first tergite more densely punctate subposteriorly (dd); second tergite more densely sculptured dorsally (ee)	***E.sardous* Guiglia, 1951**
	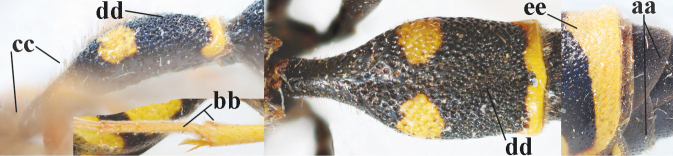	
10	Middle of propodeum with small interspaces between coarse punctures micro-sculptured (a); mesoscutum with pair of yellow (and often large) patches antero-laterally (b); clypeus with coarser punctures, especially apically (c); length of fore wing 10–13 mm	***E.punctaticlypeus* Giordani Soika, 1943**
	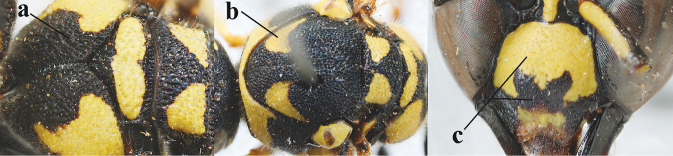	
–	Middle of propodeum either without interspaces between punctures (aa) or small interspaces present and smooth (aaa); mesoscutum entirely black or with smaller linear patches antero-laterally (bb); clypeus often only punctulate or with less coarse punctation, especially apically (cc); length of fore wing 7–11 mm	**11**
	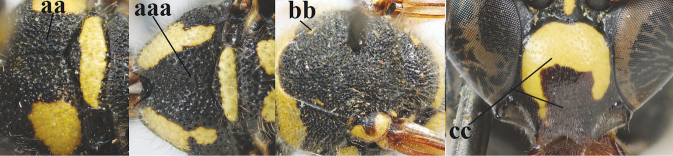	
11	Apical half of scape anteriorly (a) and clypeus (b) black or largely so; second sternite with narrow yellow band apically (c)	**12**
	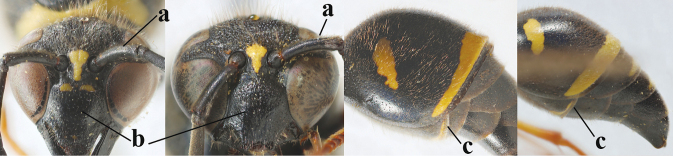	
–	Apical half of scape anteriorly (aa) usually and clypeus (bb) partly yellow; second sternite with wider yellow band apically (cc)	**13**
	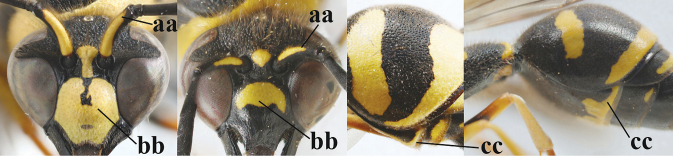	
12	Pronotum flattened medio-posteriorly and narrowly yellow (a); apical corners of clypeus more protruding (b); third metasomal tergite entirely black (c); inner tooth of hind tarsal claws shorter and wider (d) and claw curved (e); [if second sternite with some long setae and first tergite comparatively slender, see *E.coronatus*]	***E.coarctatuscoarctatus* (Linnaeus, 1758)**
	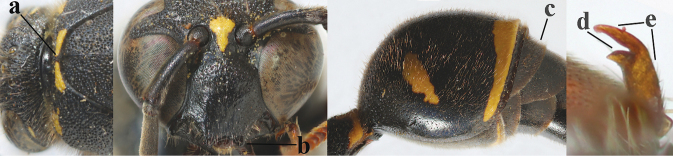	
–	Pronotum convex medio-posteriorly and wider yellow (aa); apical corners of clypeus less protruding (bb); third tergite partly yellow (cc); inner tooth of hind tarsal claws slightly longer and narrower (dd) and claw less curved (ee); [if outer side of hind coxa mostly with short setae, shape of hind claws different, second tergite more punctate and fore tibia entirely yellow, compare with very similar *E.coarctatuslunulatus* with darkened scape]	***E.pedunculatus* (Panzer, 1799)**
	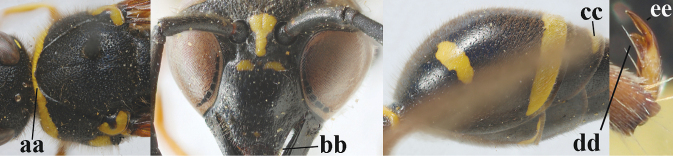	
13	Clypeus yellow medio-dorsally (a) and apical half largely black (b); posterior half of first tergite usually more robust in dorsal view (c); medio-posteriorly third sternite yellow (d); apical corners of clypeus slightly less protruding (e)	**14**
	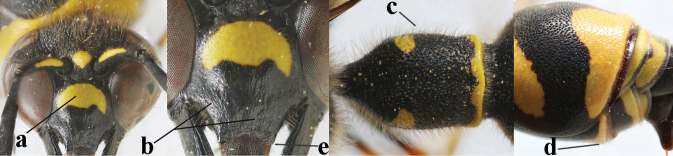	
–	Clypeus black medio-dorsally (aa), if yellow (aaa) then apical half of clypeus also largely yellow (bb); posterior half of first tergite less robust in dorsal view (cc); medio-posteriorly third sternite usually black (dd) or yellow band reduced; apical corners of clypeus slightly more protruding (ee)	**15**
	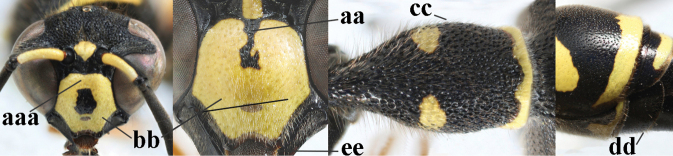	
14	Medio-posteriorly third metasomal sternite broadly black or dark brown (a); outer side of hind coxa mostly with very long setae in dorsal view (b); first tergite comparatively convex in lateral view (c); head conspicuously long setose (d); fore tibia often with dark brown or blackish patch medio-posteriorly (e); second tergite finely punctate (f); [yellow dorsal part of clypeus transverse	***E.coarctatuscoarctatus* (Linnaeus, 1758)**
	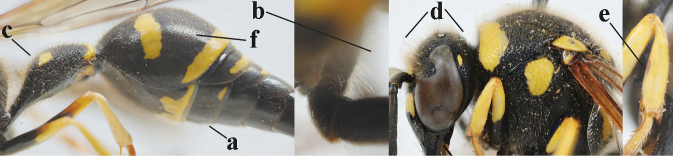	
–	Medio-posteriorly third sternite yellow (aa) or narrowly interrupted; hind coxa with short to medium-sized setae in dorsal view (bb); first tergite less convex in lateral view (cc); head less conspicuously setose (dd); fore tibia entirely yellow (ee) or with blackish patch medio-posteriorly; second tergite more coarsely punctate (ff)	***E.coarctatuslunulatus* Fabricius, 1804**
	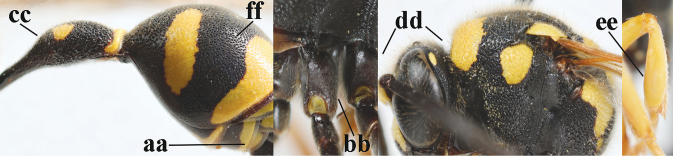	
15	Laterally propleuron regularly short setose in lateral view, setae curved and 0.1–0.3 times as long as occipital setae (a); hind tarsus (except dark brown telotarsus) brownish yellow (b); [apical lamellae of propodeum more or less darkened]	***E.pomiformis* (Fabricius, 1781)**
	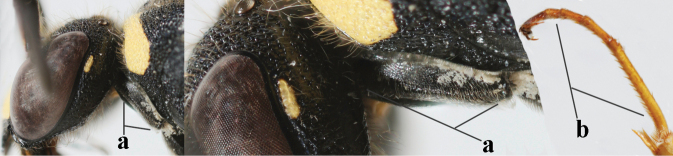	
–	Laterally propleuron irregularly medium-sized setose in lateral view, setae straight (aa) or adpressed; hind tarsus dark brown (bb) or largely so	***E.subpomiformis* Blüthgen, 1938**
	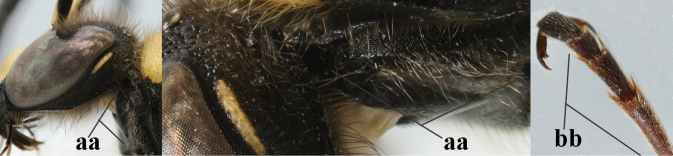	

Males

**Table:** 

16	Setae of occiput short to medium-sized (a); second and third antennal segments reddish or brownish ventrally (b); [antennal hook distinctly bent; laterally pronotum often broadly yellow]	**17**
	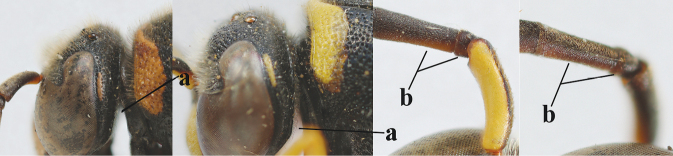	
–	Setae of occiput long (aa; but sometimes worn off); second and third antennal segments black ventrally (bb)	**19**
	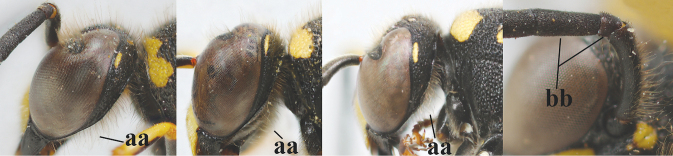	
17	Antennal hook robust (a, b) and in lateral view claw-like (b); ventro-lateral corners of clypeus rather acute (c); [first tergite bicoloured ventrally; meso­scutal setosity short]	***E.dubius* de Saussure, 1852**
	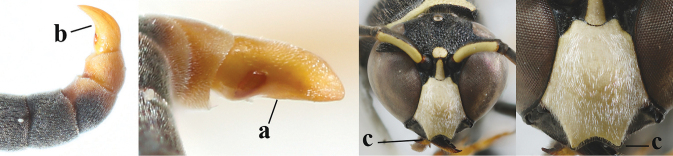	
–	Antennal hook more slender (aa, bb) and less claw-like in lateral view (bb), but sometimes intermediate in SW Europe; ventro-lateral corners of clypeus slightly more obtuse (cc); [first tergite tri- or bicoloured ventrally]	**18**
	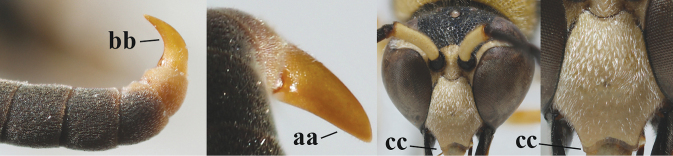	
18	First metasomal segment tricoloured ventrally (a); antennal hook more slender in lateral view (b; sometimes less than shown); setae of eye incision at most half as long as apical width of scape in lateral view (c); parategula largely yellow (d), rarely dark brown; [antenna extensively yellowish brown ventrally as in *E.dubiuspalaestinensis* Blüthgen, 1938]	***E.cyrenaicus* Blüthgen, 1938**
	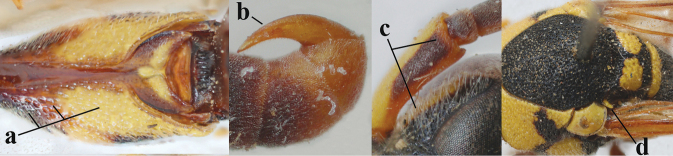	
–	First segment bicoloured ventrally (aa); antennal hook less slender in lateral view (bb); setae of eye incision about as long as apical width of scape in lateral view (cc); parategula black (dd); [**if** antennal hook minute and nearly straight, cf. *E.pomiformis* with short occipital setae]	***E.sareptanus* André, 1884**
	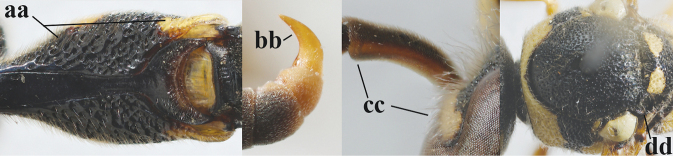	
19	Apical half of first metasomal segment largely orange or yellow dorsally (a); mesoscutum with medium-sized to large yellow or orange patch laterally (b); [propleuron with short setae]	***E.tripunctatus* (Christ, 1791)**
	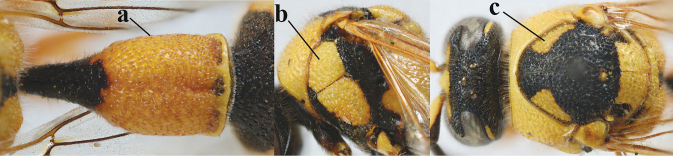	
–	Apical half of first segment largely black dorsally (aa); mesopleuron mainly or entirely black anteriorly (bb); mesoscutum black (cc) or with small transverse patch laterally (ccc), rarely larger	**20**
	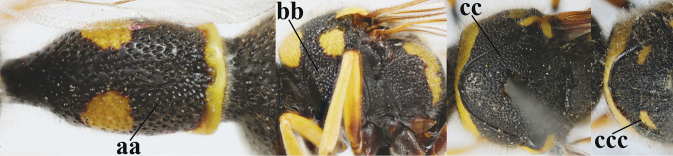	
20	Middle of propodeum with matt, micro-sculptured interspaces (a); mesoscutum with pair of transverse yellow patches antero-laterally (b), but sometimes reduced; apical antennal hook robust apically and dark brown (c); base of mandible often partly pale yellowish (d); length of fore wing 9–13 mm; [posterior half of first tergite very robust]	***E.punctaticlypeus* Giordani Soika, 1943**
	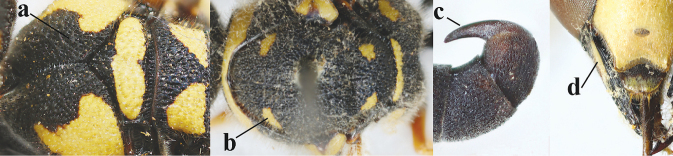	
–	Middle of propodeum without micro-sculptured interspaces (aa); mesoscutum entirely black (bb); apical antennal hook slender apically and yellow (cc), rarely infuscate or dark brown (*E.subpomiformis*); base of mandible black (dd), rarely with small yellow patches; length of fore wing 7–11 mm	**21**
	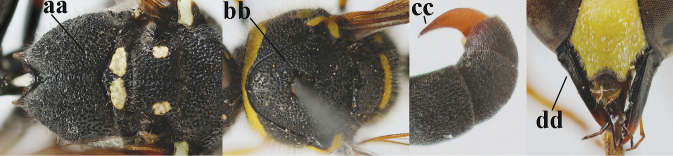	
21	Second metasomal sternite with some long setae (a), rarely medium-sized; posterior part of first tergite flattened in lateral view (b); scape black anteriorly or largely so (NW Europe; c), but more or less yellow in S Europe; ventro-apical corners of clypeus narrower (d); first tergite without pair of yellow spots (e) or spots minute; [second tergite conspicuously setose and remotely sculptured]	**22**
	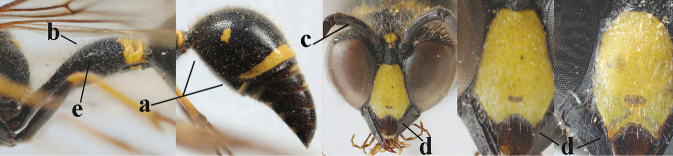	
–	Second sternite with short or medium-sized setae only (aa); posterior part of first tergite convex in lateral view (bb); scape yellow anteriorly (c) or largely so; ventro-apical corners of clypeus wider (d); usually first tergite with pair of yellow spots (ee), but often reduced in *E.papillarius*	**23**
	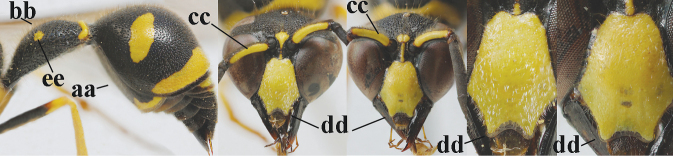	
22	Second tergite coarser punctate (a); first tergite slightly less robust in ventral view (b); third tergite more extensively yellow (c); setosity of scape either long dorsally (d) or (Balkan Peninsula) short	***E.coronatus* (Panzer, 1799)**
	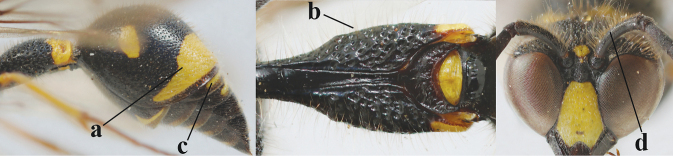	
–	Second tergite comparatively finely punctate (aa); first tergite slightly more robust in ventral view (bb); third tergite less extensively yellow (cc); scape short setose dorsally (dd); [dark form with dark scape, more or less partly long setose second sternite and finely punctate second tergite]	***E.c.coarctatus* (Linnaeus, 1758)**
	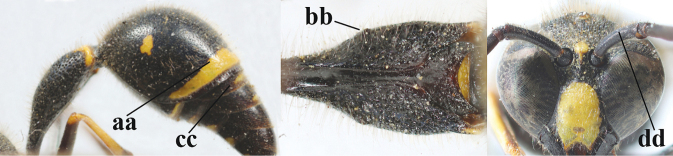	
23	Antennal hook short, bent and slightly wider medially (a); apical lamella of second tergite subhyaline or pale yellow (b); second tergite more or less concave medio-posteriorly in lateral view (c); clypeus slightly more emarginated medio-apically (d)	***E.mediterraneus* Kriechbaumer, 1879**
	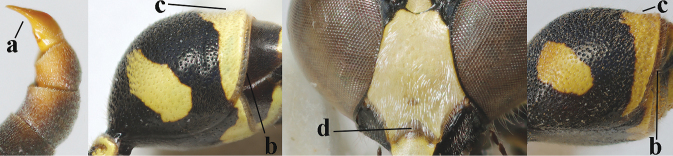	
–	Antennal hook longer, nearly straight (but medio-dorsally slightly depressed) and medially more slender (aa), but intermediate in *E.subpomiformis*; apical lamella of second tergite dark brown or blackish (bb), but yellow in *E.sardous*; second tergite flat or weakly concave medio-posteriorly in lateral view (cc); clypeus less emarginated medio-apically (dd)	**24**
	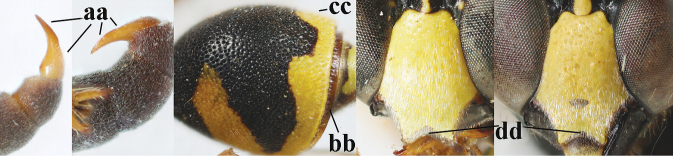	
24	Outer side of hind tibia with black or dark brown patch dorso-apically, contrasting with mainly pale hind basitarsus (a); first tergite comparatively slender in ventral (b) and dorsal (c) view; apical half of antennal hook with more or less dark brown keel dorsally and relatively slender (d); [second metasomal tergite shiny apico-laterally]	***E.papillarius* (Christ, 1791)**
	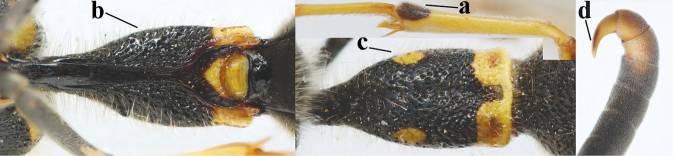	
–	Outer side of hind tibia dorso-apically and hind basitarsus yellow (aa); first tergite usually more robust in ventral (bb) and dorsal (cc) view; apical half of antennal hook without dark brown keel dorsally (dd), if slightly developed then less slender; [second tergite usually with satin sheen apico-laterally]	**25**
	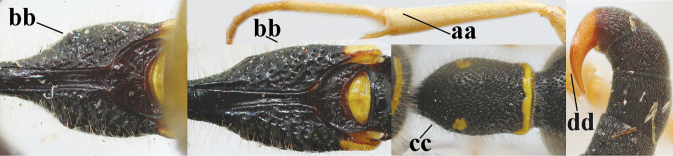	
25	Third and fourth metasomal tergites black (a), rarely third tergite narrowly yellow; mesoscutum scrobiculate-reticulate (b); apical lamella of second tergite yellow or brownish (c); [antennal hook with submedial depression]	***E.sardous* Guiglia, 1951**
	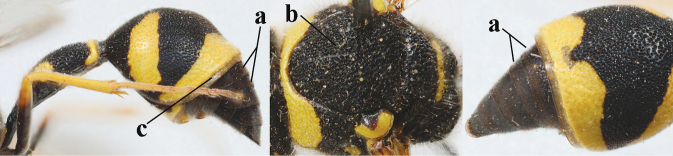	
–	Third and fourth tergites partly yellow (aa); mesoscutum coarsely punctate or punctate-reticulate (bb); apical lamella of second tergite dark brown or blackish (cc)	**26**
	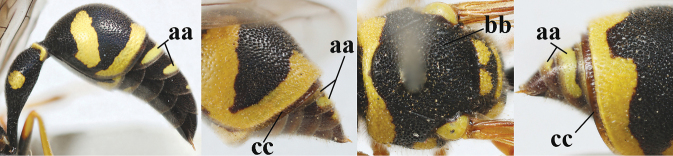	
26	Setae of propleuron regular and 0.1–0.3 times as long as occipital setae (a); antennal hook nearly straight (a; sometimes slightly more curved than illustrated)	***E.pomiformis* (Fabricius, 1781)**
	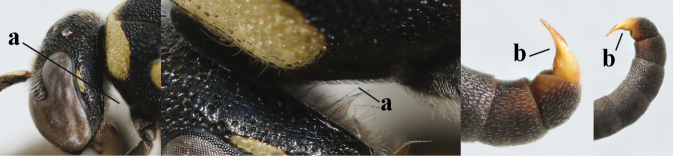	
–	Setae of propleuron irregular and mainly straight and 0.3–0.7 times as long as occipital setae (aa); antennal hook more curved (bb)	**26**
	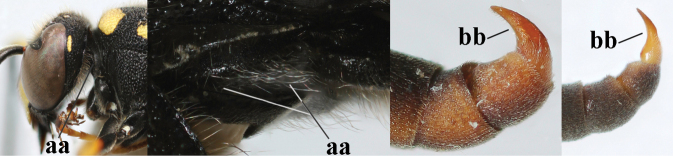	
27	Antennal hook largely dark brown or brown (a), more slender and slightly bent (b); penultimate antennal segment dark brown ventrally (c); second metasomal tergite usually rather bristly setose (d)	***E.subpomiformis* Blüthgen, 1938**
	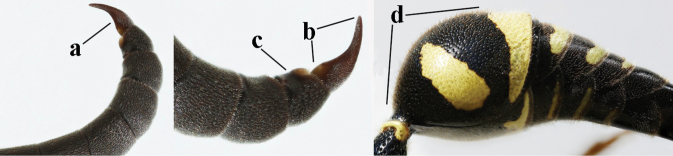	
–	Antennal hook yellow (aa) or largely so, less slender and more bent (bb); penultimate antennal segment yellowish brown ventrally (cc); second metasomal tergite usually only short setose (dd), but bristly in typical *E.coarctatus* (ddd)	**27**
	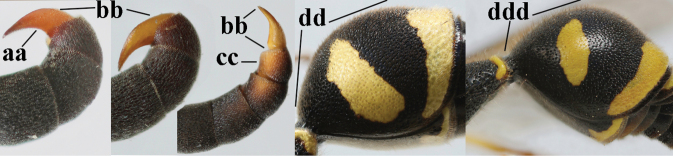	
28	Outer side of hind coxa with short to medium-sized setae in dorsal view (a); first metasomal tergite usually slightly less convex in lateral view (b); head moderately setose (c); apical yellow band of second tergite less widened dorsally (d)	***E.coarctatuslunulatus* Fabricius, 1804**
	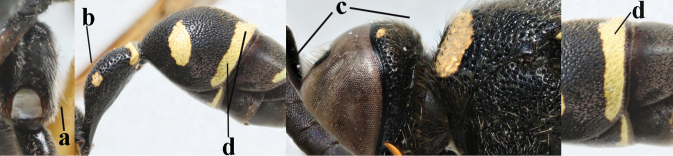	
–	Outer side of hind coxa with very long setae in dorsal view (aa); first tergite comparatively convex in lateral view (bb); head conspicuously long setose (cc); apical yellow band of second tergite often comparatively wide dorsally (dd)	**29**
	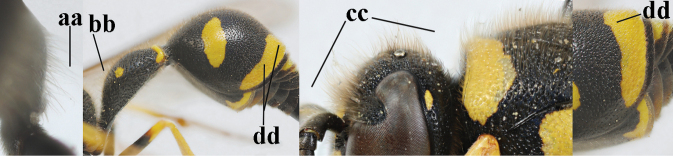	
29	Second metasomal tergite moderately to coarsely punctate (a) and usually rather dull (b); inner side of apical hook with small setae (c); [pale form of *E.coarctatus* with more or less coarsely punctate second tergite, long setose hind coxae and short setose second sternite; f. barbatulus Blüthgen, 1943]	***E.coarctatuscoarctatus* (Linnaeus, 1758)**
	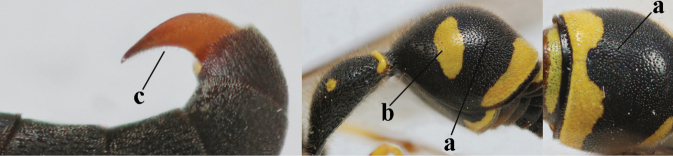	
–	Second metasomal tergite sparsely punctate or punctulate (aa) and shiny (bb); inner side of apical hook without distinct setae (cc)	***E.pedunculatus* (Panzer, 1799)**
	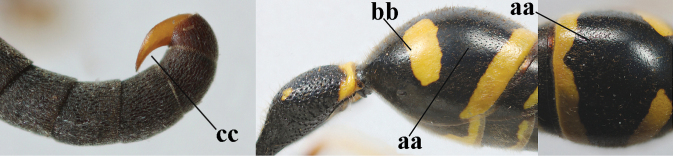	

### ﻿Species accounts

#### ﻿ Eumenes c. coarctatus

Taxon classificationAnimaliaHymenopteraVespidae

(Linnaeus, 1758)

A0B37477-DE72-51A2-B5D6-A3E0B9847D6A

Figs 2a , 4–12 , 13–21

Vespa coarctata Linnaeus, 1758: 573.
Eumenes coarctatus coarctatus ; Gusenleitner, 1972: 75, 1999: 569; Day 1979: 60; Richards 1980: 21; Vergés Serra 1985: 145, 149; Castro 1997: 4; Sanza 1997: 435, 445; Giordani Soika and Borsato 1995: 6; Borsato 2006: 140–141; Castro and Sanza 2009: 265; Frommer 2013: 19.
Eumenes coarctatus ; Tobias and Kurzenko 1978: 160; Richards 1980: 20–21; Hensen 1985: 45; Schmid-Egger and Schmidt 2002: 18; Archer 2003: 64, 2014: 32; Smit et al. 2004: 326–327; Woydak 2006: 39–40; Schmid-Egger 2010: 23, 2011: 44; Abenius 2012: 271; Neumeyer 2014: 367, 2019: 269; Fateryga et al. 2020: 95; Baldock et al. 2020: 43; Cassar et al. 2022: 206–207.
Eumenes (Eumenes) coarctatus ; Fateryga 2017: 181, 2018: 203–204.
Eumenes (Eumenes) coarctatus coarctatus ; van der Vecht and Fischer 1972: 125–126 (literature before 1972); Castro 1997: 4; Gereys 2006: 387, 2012: 200, 2016: 127; Dal Pos et al. 2022: 14.
Eumenes pomiformis dernaensis Blüthgen, 1938: 494; Fateryga 2017: 181 (as synonym of E.coarctatus).
Eumenes lunulatus dernaensis ; Gusenleitner 1972: 80; van der Vecht and Fischer 1972: 128.
Eumenes coarctatus dernansis (!); Gusenleitner 1999: 569.
Eumenes coarctatus dernaensis ; Gusenleitner 2013: 26.
Eumenes pomiformis barbatulus Blüthgen, 1943: 303; Gusenleitner 1972: 75 (as synonym of E.coarctatus); van der Vecht and Fischer 1972: 125 (id.); Fateryga 2017: 181 (id.).
Eumenes pedunculata var. punctata Hellén, 1944: 11; van der Vecht and Fischer 1972: 125 (as synonym of E.coarctatus); Gereys 2016: 127 (id.); Fateryga 2017: 181 (id.).
Eumenes pedunculatus var. turaniformis Blüthgen, 1959: 13; Fateryga 2017: 181 (as synonym of E.coarctatus).
Eumenes coarctatus turaniformis ; Gusenleitner 1972: 76, 1999: 569.
Eumenes (Eumenes) coarctatus turaniformis ; van der Vecht 1968: 78; van der Vecht and Fischer 1972: 126; Dal Pos et al. 2022: 14.
Eumenes coarctatus corsicus Gusenleitner, 1972: 77, 1999: 569 (as synonym of E.coarctatus); Borsato 2006: 141; Gereys 2016: 127 (id.); Fateryga 2017: 181 (id.).
Eumenes coarctatus maroccanus Gusenleitner, 1972: 76–77, 1999: 569, 2013: 26; Castro 1997: 4 (as synonym of E.coarctatus); Gereys 2012: 200 (id.), 2016: 127 (id.); Fateryga 2017: 181 (id.).
Eumenes (Eumenes) coarctatus maroccanus ; Giordani Soika and Borsato 1995: 6; Borsato and Turrisi 2004: 143; Dal Pos et al. 2022: 14.
Eumenes coarctatus nugaricus Giordani Soika, 1986: 123; Gusenleitner 1999: 570; Borsato 2006: 141; Fateryga 2017: 181 (as synonym of E.coarctatus).
Eumenes (Eumenes) coarctatus nuragicus ; Dal Pos et al. 2022: 14.

##### Notes.

The holotype female of *E.coarctatus* is heavily damaged (e.g., antenna completely missing) but the metasoma is preserved showing the robust first tergite in lateral view (https://linnean-online.org/16751/) and the second sternite lacks long setae (see van der Vecht 1968, also for the variation of the shape of the first metasomal tergite). The clypeus is entirely black as was likely the anterior face of the scape and, therefore, belongs to the dark typical form.

##### Distribution.

The dark typical form is mostly boreo-alpine of distribution and occurs in Scandinavia (up to S Finland and SE Sweden), UK (England and Wales) and mountainous regions in Central Europe (reaching 1550 m altitude in Switzerland). The pale typical form (= f. barbatulus Blüthgen, 1943) occurs in mainly C and SW Europe and is often the most common species. Outside Europe known from N Africa and the East Palaearctic region up to China and Japan. Only breeding species in UK where it is considered a most threatened species and is known as the Heath Potter Wasp (https://naturebftb.co.uk/artwork/heath-tiger-beetle-alex-hyde/pots-of-the-heath-potter-wasp-eumenes-coarctatus/).

#### ﻿ Eumenes coarctatus lunulatus

Taxon classificationAnimaliaHymenopteraVespidae

Fabricius, 1804

19C25353-82A8-5BC4-BB7D-502E7E3CE5BE

Figs 22–30 , 31–39

Eumenes lunulata Fabricius, 1804: 290; Fateryga 2017: 181 (as synonym of E.coarctatus).
Eumenes lunulatus ; Tobias and Kurzenko 1978: 161.
Eumenes (Eumenes) lunulatus lunulatus ; van der Vecht and Fischer 1972: 127 (literature before 1972); Castro 1992: 24–25, 1997: 4.
Eumenes lunulatus lunulatus ; Gusenleitner 1972: 78–79; Giordani Soika and Borsato 1995: 7; Castro and Sanza 2009: 265 (as synonym of E.coarctatus).
Eumenes coarctatus lunulatus ; Gusenleitner 1999: 565, 567, 570, 2013: 26; Schmid-Egger 2011: 44; Borsato and Turrisi 2004: 143.
Eumenes pomiformis ordubadensis Blüthgen, 1938: 493; Gusenleitner 1972: 80; Fateryga 2017: 181 (as synonym of E.coarctatus).
Eumenes (Eumenes) lunulatus ordubadensis ; van der Vecht and Fischer 1972: 129 (literature before 1972).
Eumenes lunulatus ordubadensis ; Gusenleitner 1972: 80.
Eumenes coarctatus ordubadensis ; Gusenleitner 1999: 569, 2013: 26.
Eumenes (Eumenes) coarctatus ordubadensis ; Dal Pos et al. 2022: 14.
Eumenes pomiformis limissicus Blüthgen, 1938: 493; Fateryga 2017: 181 (as synonym of E.coarctatus).
Eumenes lunulatus limissicus ; Gusenleitner 1970: 163, 1972: 80–81.
Eumenes (Eumenes) lunulatus limissicus ; van der Vecht and Fischer 1972: 128.
Eumenes coarctatus limissicus ; Gusenleitner 1999: 569, 2013: 26.
Eumenes (Eumenes) coarctatus limissicus ; Dal Pos et al. 2022: 14.
Eumenes lunulatus var. tenebricosus Gusenleitner, 1972: 79; Borsato and Turrisi 2004: 143 (as synonym of E.lunulatus); Gereys 2016: 127 (as synonym of E.coarctatus); Fateryga 2017: 181 (id.).
Eumenes lunulatus tenebricosus ; Giordani Soika and Borsato 1995: 7.
Eumenes lunulatus var. balcanicus Gusenleitner, 1972: 79; Gereys 2016: 127 (as synonym of E.coarctatus); Fateryga 2017: 181 (id.). Note. The figured female from Cyprus (Figs 22–30) is incorrectly labelled as paratype of var. balcanicus, because Cyprus was not mentioned in the original description.
Eumenes coarctatus ; Arens 2012: 489–490; Gogala 2022: 26.

##### Notes.

The holotype female of *E.lunulatus* was digitally examined by using photographs kindly supplied by Sree Gayathree Selvantharan and Lars Vilhelmsen (NHMD). Unfortunately, the head is missing, but the remaining body parts agree with the current interpretation. Gusenleitner (1972) recognised *E.lunulatus* as a valid species; however, in 1999 he lowered its rank to subspecies because of observed intermediate variation (Gusenleitner 1999). This variation in *E.coarctatus* sensu lato was one of the reasons to start this revision and thanks to the molecular data (Schmid-Egger and Schmidt 2021; this paper) some provisional conclusions can be drawn. Obviously, *Eumenescoarctatus* sensu stricto has two forms in Europe: the dark typical one in boreo-alpine Europe and a paler one in C and SW Europe, both with comparatively robust first tergite and antennal hook (Figs 20, 21). The SE (and partly C) European specimens belong to *E.coarctatuslunulatus* as defined in this paper often have a more slender first tergite (but variation is extensive) and a more slender antennal hook in the males (for lateral aspect see Fig. 37). Schmid-Egger and Schmidt (2021) did find differences in CO1 for *E.c.coarctatus* and *E.c.lunulatus*, but the latter consisted of a series from Cyprus (probably concerns Blüthgen’s ssp. limissicus) and the remainder of *E.coarctatus* originated from Germany, France, and Italy. We analysed specimens from Finland, Netherlands and Bulgaria and we found the *E.c.lunulatus* from SE Europe to be different from both the dark (Finland) and pale (Netherlands) forms of *E.c.coarctatus* (Fig. 3).

**Figures 4–12.**
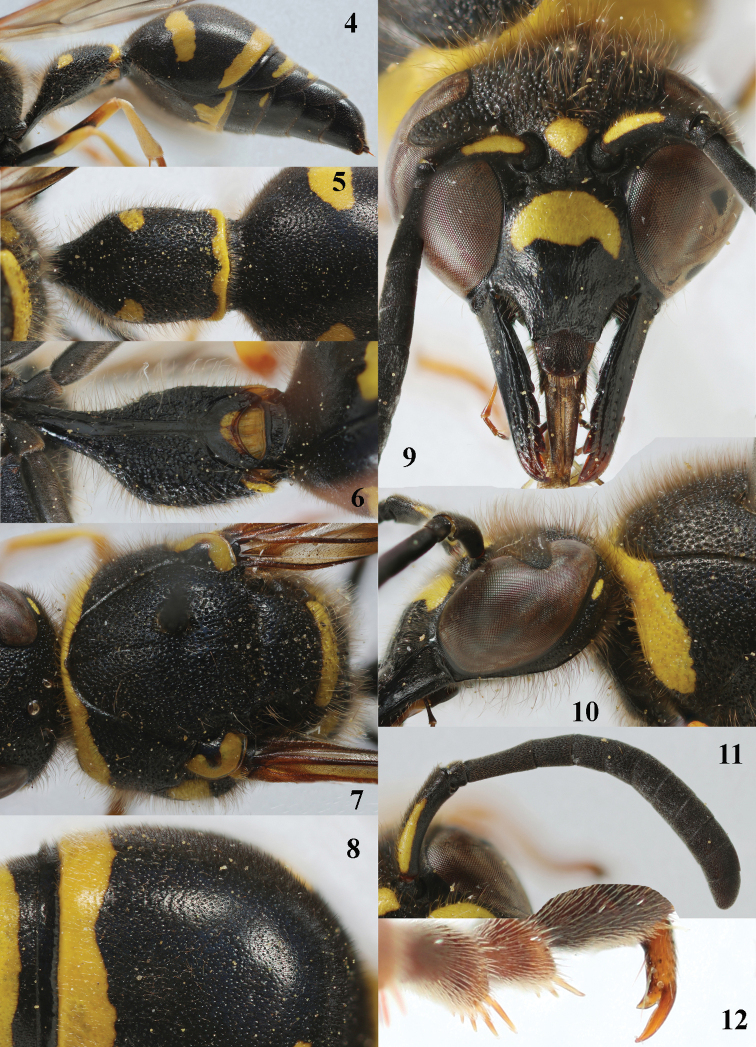
*Eumenescoarctatuscoarctatus* (Linnaeus), Netherlands (Otterloo), female **4** metasoma lateral **5** first metasomal tergite dorsal **6** first tergite ventral **7** mesosoma dorsal **8** second metasomal tergite latero-dorsal **9** head anterior **10** head and propleuron lateral **11** antenna **12** hind tarsal claw.

**Figures 13–21.**
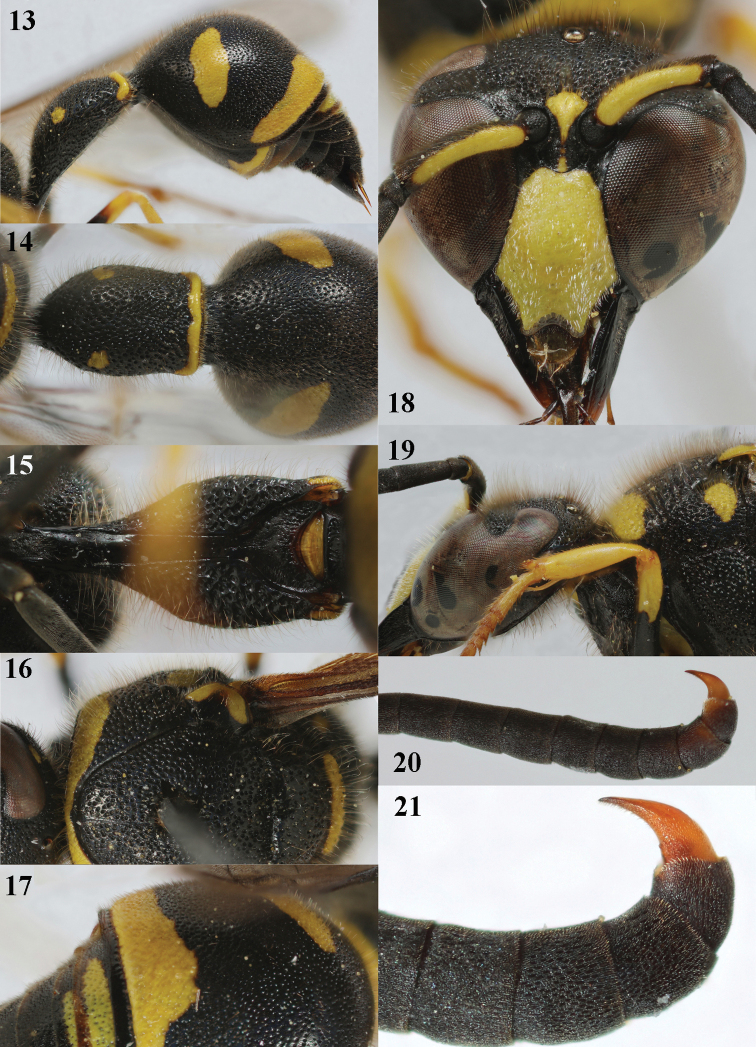
*Eumenescoarctatuscoarctatus* (Linnaeus), Netherlands (Otterloo), male **13** metasoma lateral **14** first metasomal tergite dorsal **15** first tergite ventral **16** mesosoma dorsal **17** second metasomal tergite dorsal **18** head anterior **19** head and propleuron lateral **11** apical half of antenna **12** apical hook of antenna lateral.

Neumeyer and Praz (2015) did not find the differences of COI between Swiss specimens of *E.coarctatuslunulatus* and *E.coarctatus* sensu stricto substantial enough to recognise *E.lunulatus* as separate species or subspecies. Earlier Castro and Sanza (2009) came to the same conclusion on basis of Spanish material, but *E.coarctatuslunulatus* as defined in this paper may not occur on the Iberian Peninsula. An extensive survey is needed to reveal the extent of its distribution and whether or not its status as a valid subspecies is justified or that it is just a more punctate south-eastern form of *E.coarctatus*. The yellow dorsal part of the clypeus is more or less reversed U-shaped in f. balcanicus Gusenleitner, 1972. If the clypeus is entirely yellow, middle and hind coxae with a yellow patch and the third sternite largely yellow, see f. ordubadensis Blüthgen, 1938.

**Figures 22–30.**
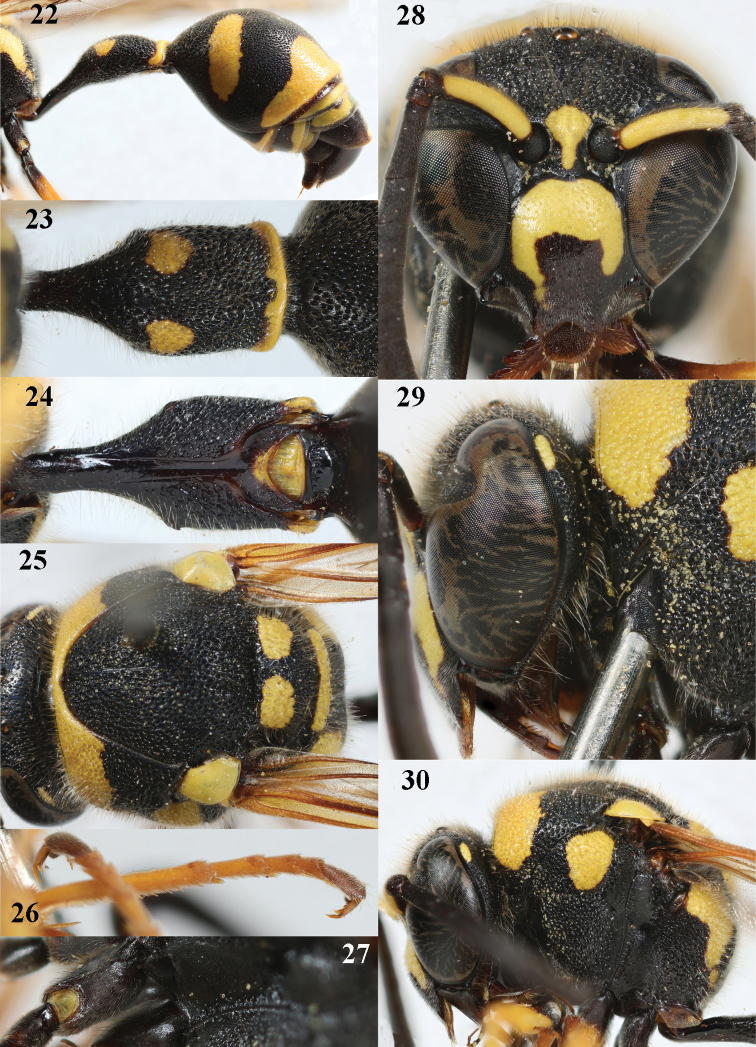
*Eumenescoarctatuslunulatus* Fabricius, Cyprus, female **22** metasoma lateral **23** first metasomal tergite dorsal **24** first tergite ventral **25** mesosoma dorsal **26** hind tarsal claw **27** hind coxa dorsal **28** head anterior **29** head and propleuron lateral **30** head and mesosoma lateral.

**Figures 31–39.**
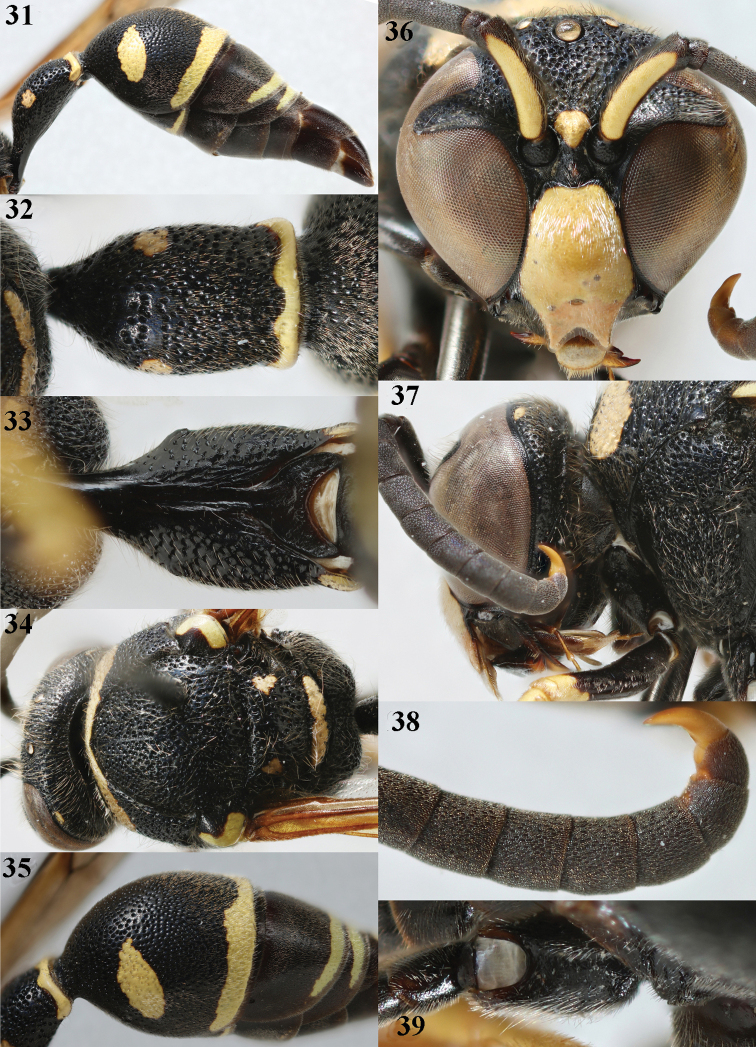
*Eumenescoarctatuslunulatus* Fabricius, Bulgaria, male **31** metasoma lateral **32** first metasomal tergite dorsal **33** first tergite ventral **34** head and mesosoma dorsal **35** second metasomal tergite dorso-lateral **36** head anterior **37** head and propleuron lateral **38** apical hook of antenna lateral **39** hind coxa dorsal.

##### Distribution.

SE and C Europe, NW Asia. Examined specimens originating from Austria (type locality), Hungary, Czech Republic, and Slovakia (but most specimens from these countries in collections belong to *E.coarctatus* sensu stricto), Italy, Bulgaria, Turkey, Greece (in Peloponnesus the most common species according to Arens (2012, listed as *E.coarctatus*), Iran.

#### ﻿ Eumenes coronatus

Taxon classificationAnimaliaHymenopteraVespidae

(Panzer, 1799)

A07DF689-5A58-5107-A1EF-38C647B3BACB

Figs 1a , 40–46 , 47–54

Vespa coronata Panzer, 1799: (6) 64: 12, pl. 12.
Eumenes (Eumenes) coronatus coronatus ; Gereys 2016: 128–129; van der Vecht and Fischer 1972: 126 (literature before 1972); Dal Pos et al. 2022: 14.
Eumenes coronatus ; Tobias and Kurzenko 1978: 160; Hensen 1985: 45; Vergés Serra 1985: 145; Castro 1997: 4; Sanza 1997: 439; ; Schmid-Egger and Schmidt 2002: 18; Schmid-Egger 2004: 72, 2010: 23; Smit et al. 2004: 327; Borsato and Turrisi 2004: 143; Woydak 2006: 40–41; Castro and Sanza 2009: 266; Abenius 2012: 270; Arens 2012: 487; Gusenleitner 2013: 26; Neumeyer 2014: 367; 2019: 270; Fateryga et al. 2020: 95; Baldock et al. 2020: 43.
Eumenes (Eumenes) coronatus ; Castro 1997: 4; Fateryga 2017: 181, 2018: 204–205.
Eumenes coronatus coronatus ; Gusenleitner 1972: 85, 1999: 570; Borsato 2006: 142; Gogala 2022: 26.
Eumenes atricornis Fabricius, 1804: 289; Gusenleitner 1972: 85 (as synonym of E.coronatus); van der Vecht and Fischer 1972: 126 (id.); Gereys 2016: 128 (id.); Fateryga 2017: 181 (id.).
Eumenes costata (!) Lucciani, 1845: CX; Fateryga 2017: 181 (as synonym of E.coronatus).
Eumenis (!) mediterraneavar.neesi Kriechbaumer, 1879: 88; Gusenleitner 1972: 85 (as synonym of E.coronatus); van der Vecht and Fischer 1972: 126 (id.); Fateryga 2017: 181 (id.).
Eumenes coarctatus var. opulenta Blüthgen, 1938: 482–483; van der Vecht and Fischer 1972: 126 (as synonym of E.coronatus); Gereys 2016: 128 (id.); Fateryga 2017: 181 (id.).
Eumenes coarctatus detonsus Blüthgen, 1943: 297; Gusenleitner 1972: 86–87, 1999: 570, 2013: 27; Fateryga 2017: 181 (as synonym of E.coronatus).
Eumenes (Eumenes) coronatus detonsus ; van der Vecht and Fischer 1972: 127; Dal Pos et al. 2022: 15.
Eumenes coarctatus ab. nigrotibia Hellén, 1944: 11; van der Vecht and Fischer 1972: 126 (as synonym of E.coronatus); Gereys 2016: 128 (id.); Fateryga 2017: 181 (id.).
Eumenes coarctatus var. niger Szulczewski, 1950: 8 (invalid homonym; as synonym of E.coronatus); Gereys 2016: 128 (id.); Fateryga 2017: 181 (id.).
Eumenes coarctatus ibericus Blüthgen, 1956: 2; Gusenleitner 1972: 85, 1999: 571; van der Vecht and Fischer 1972: 127; Castro 1997: 4 (as synonym of E.coronatus); Gereys 2016: 128 (id.); Fateryga 2017: 181 (id.).
Eumenes coronatus corruetus Gusenleitner, 1972: 87, 1999: 570–571, 2013: 26; Fateryga 2017: 181 (as synonym of E.coronatus).
Eumenes (Eumenes) coronatus corruetus ; Dal Pos et al. 2022: 15.

##### Notes.

Males from the Balkan Peninsula have the apical half of antennal hook distinctly flattened, different from the wider apical half in Central European males (Fig. 54).

**Figures 40–46.**
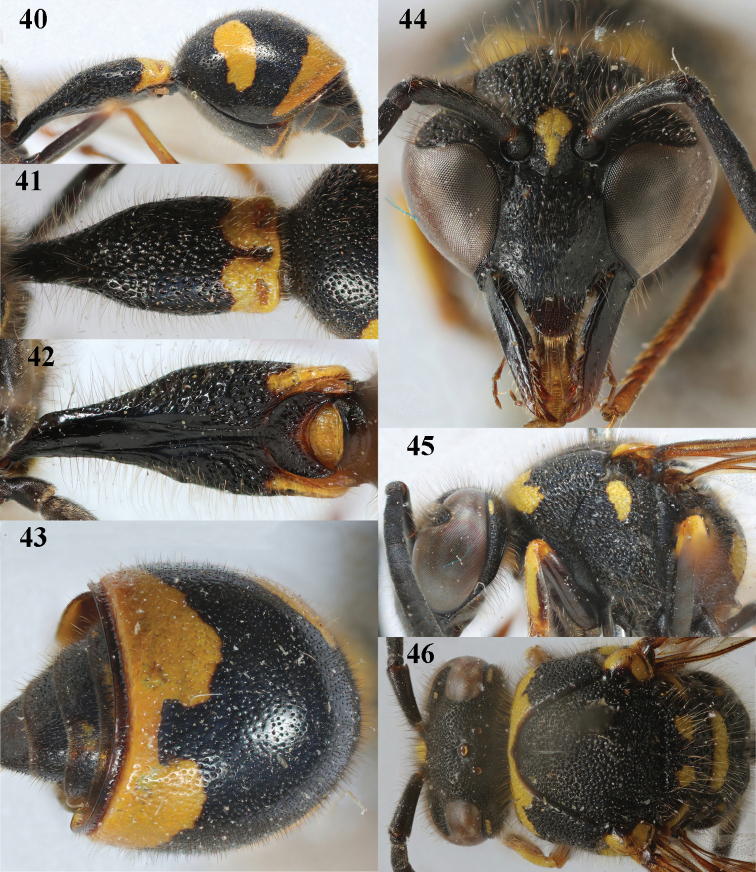
*Eumenescoronatus* (Panzer), France, female **40** metasoma lateral **41** first metasomal tergite dorsal **42** first tergite ventral **43** second metasomal tergite dorsal **44** head anterior **45** head and mesosoma lateral **46** head and mesosoma dorsal.

##### Distribution.

Rather common in most of Europe (including southern Scandinavia; Abenius 2012), but absent in the UK. In Switzerland up to 1640 m altitude, but in Peloponnesus (S Greece) not found above 1200 m altitude (Arens 2012). Outside Europe in Israel, Turkey, Iran and in East Palaearctic region up to Mongolia, China, and Korea.

**Figures 47–54.**
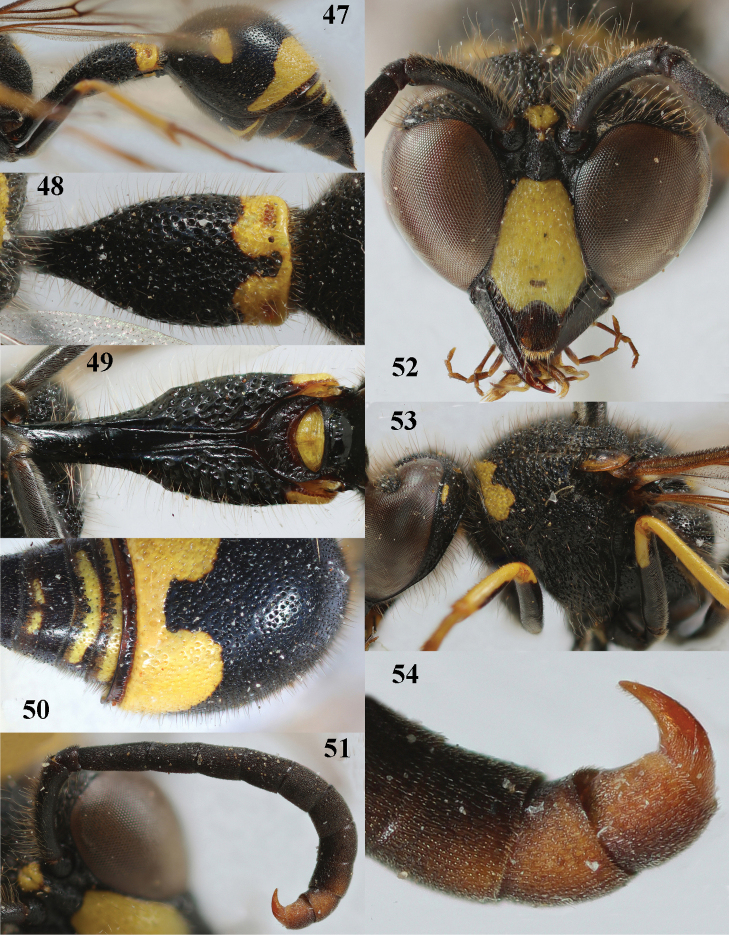
*Eumenescoronatus* (Panzer), Spain, male **47** metasoma lateral **48** first metasomal tergite dorsal **49** first tergite ventral **50** second metasomal tergite dorsal **51** antenna anterior **52** head anterior **53** head and mesosoma lateral **54** apical hook of antenna lateral.

**Figures 55–64.**
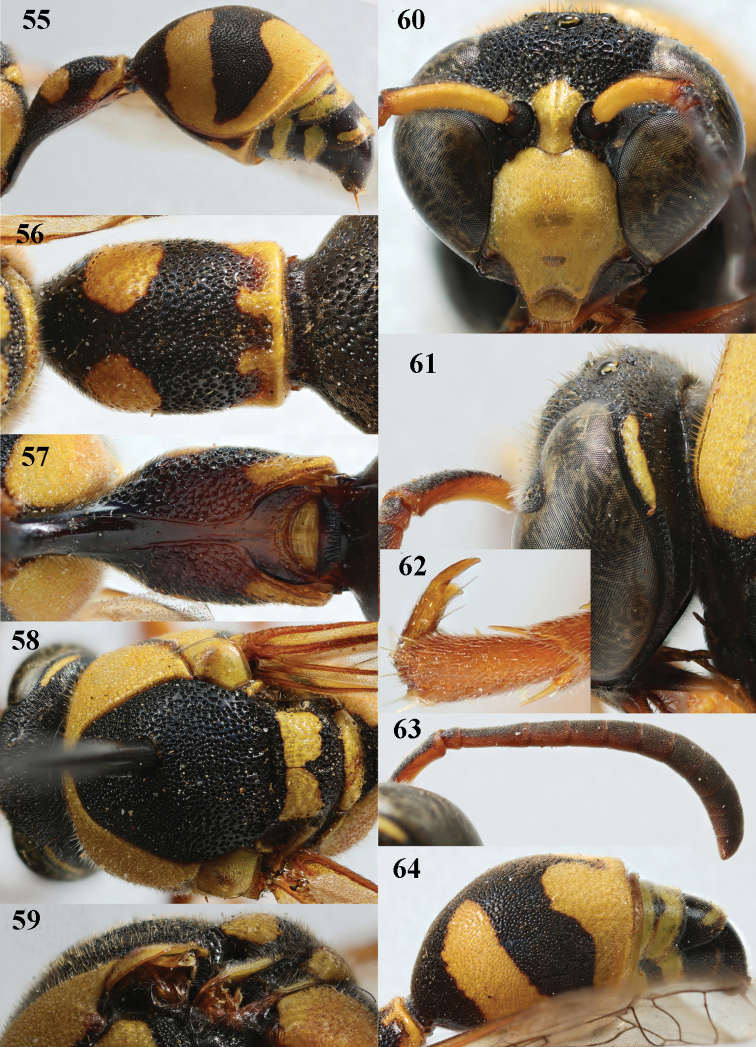
*Eumenescyrenaicus* Blüthgen, Italy (Sardinia), female **55** metasoma lateral **56** first metasomal tergite dorsal **57** first tergite ventral **58** mesosoma dorsal **59** mesoscutum and scutellum lateral **60** head anterior **61** head and propleuron lateral **62** hind tarsal claw **63** antenna **64** second metasomal tergite dorso-lateral.

**Figures 65–74.**
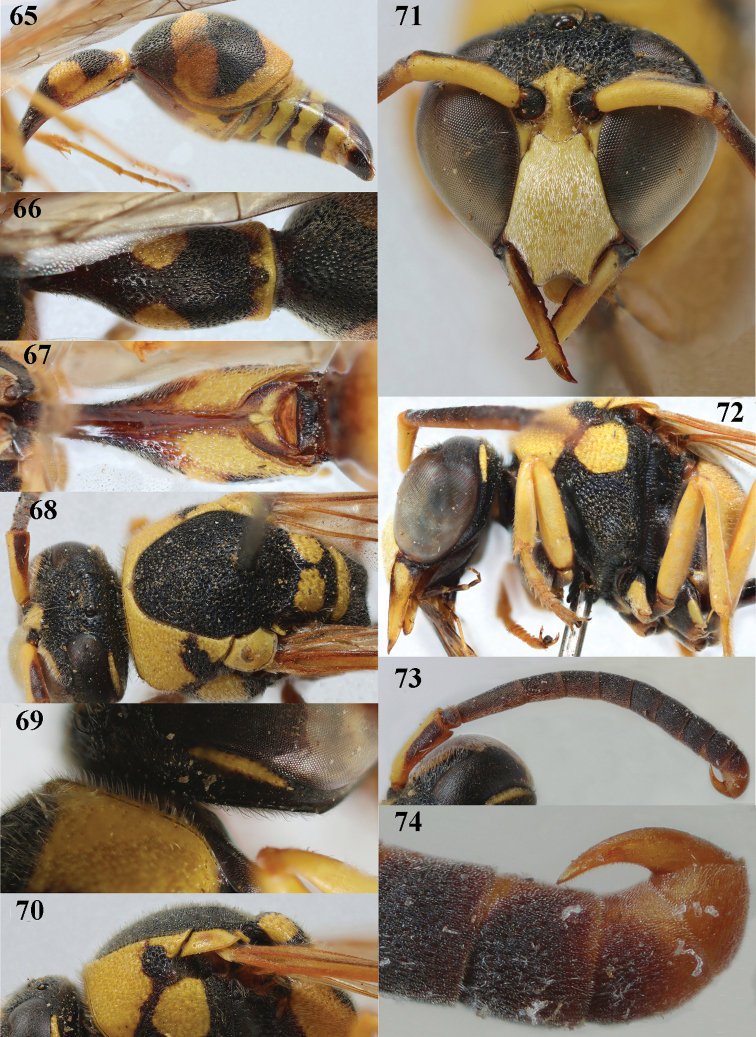
*Eumenescyrenaicus* Blüthgen, Morocco, male **65** metasoma lateral **66** first metasomal tergite dorsal **67** first tergite ventral **68** head and mesosoma dorsal **69** head posteriorly and propleuron lateral **70** mesoscutum and scutellum lateral **71** head anterior **72** head and mesosoma lateral **73** antenna **74** apical hook of antenna lateral.

#### ﻿ Eumenes cyrenaicus

Taxon classificationAnimaliaHymenopteraVespidae

Blüthgen, 1938

22A1A356-A228-5D94-AA43-DCEF05405521

Figs 55–64 , 65–74

Eumenes dubius cyrenaicus Blüthgen, 1938: 464, 468.
Eumenes (Eumenes) dubius cyrenaicus ; van der Vecht and Fischer 1972: 127 (literature before 1972).
Eumenes (Eumenes) cyrenaicus cyrenaicus ; Dal Pos et al. 2022: 15.
Eumenes dubius cyrenaicus var. congruens Blüthgen, 1938: 464; van der Vecht and Fischer 1972: 127 (as synonym of E.cyrenaicus).
Eumenes dubius dubius var. pseudogermanica Blüthgen, 1938: 464.
Eumenes (Eumenes) dubius pseudogermanicus ; van der Vecht and Fischer 1972: 127.
Eumenes cyrenaicus pseudogermanicus ; Giordani Soika and Borsato 1995: 7; Gusenleitner 1999: 571, 2013: 27; Borsato and Turrisi 2004: 144; Borsato 2006: 142.
Eumenes (Eumenes) cyrenaicus pseudogermanicus ; Dal Pos et al. 2022: 15.

##### Notes.

*Eumenescyrenaicus* is similarly coloured as E.dubiusf.palaestinensis Blüthgen, 1938 from Asia Minor, but *E.cyrenaicus* has the yellow stripe of the eye incision narrow or absent (wide in E.dubiusf.palaestinensis) and the clypeus sparser setose (densely silvery setose in E.dubiusf.palaestinensis). Males can be separated by the shape of the antennal hook (in ventral view normal in *E.cyrenaicus* and widened in E.dubiusf.palaestinensis) and sculpture of fifth sternite (distinctly punctate in *E.cyrenaicus* and punctulate in E.dubiusf.palaestinensis). Typical E.dubiusf.palaestinensis has the apical lamella of the second tergite yellow and in *E.cyrenaicus* light brown or yellowish (Fig. 65); Sardinia, Spain and Portugal, N Africa) to blackish brown (Italy, N Macedonia, Greece, Bulgaria, Turkey, but sometimes also pale brown (Fig. 55); Gusenleitner (1972) already mentioned the variability of this character for *E.dubius* in Asia. The separation of *E.cyrenaicus* from *E.sareptanus* is mostly based on colour differences and, therefore, may be problematic. In general, females of *E.cyrenaicus* have a more robust first tergite, including the petiolate part and males have the antennal hook more curved than in *E.sareptanus*.

##### Distribution.

North Africa, South Europe (*Spain, *Portugal, Italy (Sardinia, Sicily), *N Macedonia, *Bulgaria, *Greece) and *Turkey.

#### ﻿ Eumenes dubius

Taxon classificationAnimaliaHymenopteraVespidae

de Saussure, 1852

85C77FBA-BB80-52D6-B05E-7FA8821C1B0B

Figs 75–84 , 85–93

Eumenes dubia de Saussure, 1852: 32 (depository of type series unknown).
Eumenes dubius ; Tobias and Kurzenko 1978: 160; Giordani Soika and Borsato 1995: 7; Arens 2012: 486; Neumeyer 2019: 275; Baldock et al. 2020: 43; Cassar et al. 2022: 207.
Eumenes dubius dubius ; Castro 1997: 4; Borsato and Turrisi 2004: 144; Borsato 2006: 142; Castro and Sanza 2009: 266; Gusenleitner 2013: 27.
Eumenes (Eumenes) dubius dubius ; van der Vecht and Fischer 1972: 127 (literature before 1972); Castro 1992: 24; Dal Pos et al. 2022: 15.
Eumenes (Eumenes) dubius ; Fateryga 2017: 181–182, 2018: 206.
Eumenes (Eumenes) dubius dubius var. palaestinensis Blüthgen, 1938: 467; Fateryga 2017 (as synonym of E.dubius).
Eumenes (Eumenes) dubius palaestinensis ; van der Vecht and Fischer 1972: 127 (literature before 1972); Dal Pos et al. 2022: 15.
Eumenes (Eumenes) dubius dubius var. macedonica Blüthgen, 1952: 5, 15; Fateryga 2017 (as synonym of E.dubius).
Eumenes (Eumenes) dubius macedonicus ; van der Vecht and Fischer 1972: 127 (literature before 1972); Dal Pos et al. 2022: 15.

##### Notes.

Rarely collected species in C and S Europe, but common in Spain and Portugal (Castro 1992; Baldock et al. 2020) and S Greece (Arens 2012). Castro (1992) collected a large series in NE Spain and because of the observed variation in his series he concluded that the European specimens of *E.sareptanus* should be included under *E.dubius*. According to the NJ tree based on COI sequences in Schmid-Egger and Schmidt (2021)*E.sareptanus* is a species separate from *E.dubius* (both from Spain) and both differ from *E.dubius* from Cyprus. According to Gusenleitner (1972) specimens from Cyprus are more or less intermediately coloured to *E.d.palaestinensis* Blüthgen and possibly this name should be applied if it concerns a valid species. Because of the differences in COI sequences combined with the usually distinct difference in the shape of the male apical antennal segment we refrain from including *E.sareptanus* under *E.dubius* as was proposed by Castro (1992), Gereys (2016) and Baldock et al. (2020) till a more thoroughly study is made of this complex. Traditionally, *E.dubius* is separated from *E.sareptanus* by having the setae of the mesoscutum about half as long as apical width of the scape and the apical lamella of the second tergite as long as height of preapical vertical depression of the tergite. Unfortunately, both seems too variable for separating both taxa.

**Figures 75–84.** *Eumenesdubius* de Saussure, Bulgaria, female **75** metasoma lateral **76** first metasomal tergite dorsal **77** first tergite ventral **78** mesosoma dorsal **79** second metasomal tergite latero-dorsal **80** head anterior **81** head and propleuron lateral **82** antenna **83** hind tarsal claws **84** mesoscutum and scutellum lateral.

**Figures 85–93.**
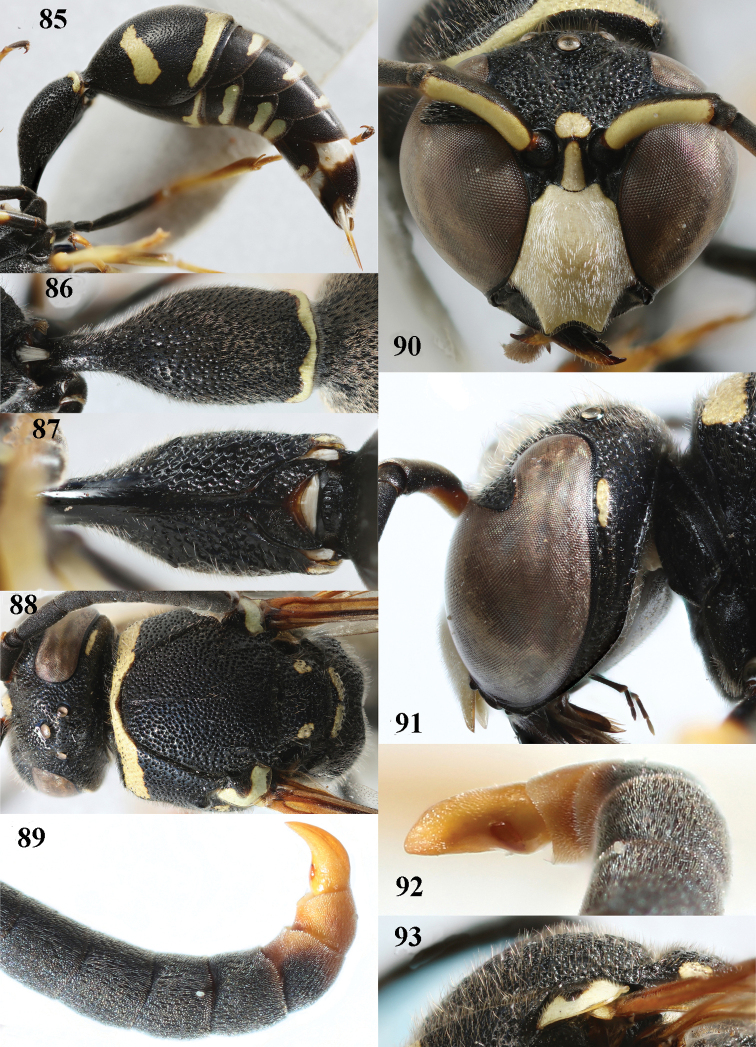
*Eumenesdubius* de Saussure, Bulgaria, male **85** metasoma lateral **86** first metasomal tergite dorsal **87** first tergite ventral **88** head and mesosoma dorsal **89** apical hook of antenna lateral **90** head anterior **91** head and propleuron lateral **92** apical hook of antenna latero-ventral **93** mesoscutum and scutellum lateral.

##### Distribution.

Asia, Central and South Europe. Absent in Switzerland according to Neumeyer (2019) and common in Peloponnesus (Greece; Arens 2012). Outside Europe in N Africa, Caucasus, Turkey, Syria, Jordan, Lebanon, Israel, Iraq, Iran, Turkmenistan, Tajikistan, and Kazakhstan. Introduced in S America (Gusenleitner 1999).

#### ﻿ Eumenes mediterraneus

Taxon classificationAnimaliaHymenopteraVespidae

Kriechbaumer, 1879 sensu lato

380F5E57-0572-5084-8317-02C702A2202D

Figs 94–102 , 103–111

Eumenis (sic!) mediterraneus Kriechbaumer, 1879: 85.
Eumenes (Eumenes) mediterraneus mediterraneus ; van der Vecht and Fischer 1972: 129 (literature before 1972); Castro 1992: 25, 1997: 4; Dal Pos et al. 2022: 15.
Eumenes (Eumenes) mediterraneus ; Frommer 2012: 176–182; Fateryga 2017: 182, 2018: 206–207.
Eumenes mediterraneus ; Tobias and Kurzenko 1978: 160; Giordani Soika and Borsato 1995: 7; Arens 2012: 488; Neumeyer 2014: 367; 2019: 271; Baldock et al. 2020: 43; Cassar et al. 2022: 207.
Eumenes mediterraneus mediterraneus ; Borsato and Turrisi 2004: 144; Borsato 2006: 142–143; Castro and Sanza 2009: 266; Gusenleitner 2013: 28.
Eumenes affinissima race quettaensis Cameron, 1907: 132–133; Fateryga 2017: 182 (as synonym of E.mediterraneus).
Eumenes (Eumenes) mediterraneus quettaensis ; van der Vecht and Fischer 1972: 129 (literature before 1972); Dal Pos et al. 2022: 15.
Eumenes (Eumenes) mediterraneus quettaensis ; Gusenleitner 2013: 28.
Labus superbus Meade-Waldo, 1910: 36; Fateryga 2017: 182 (as synonym of E.mediterraneus).
Eumenes (Eumenes) mediterraneus superbus ; van der Vecht and Fischer 1972: 130 (literature before 1972).
Eumenes mediterraneus bengasinus Blüthgen, 1938: 487; Gusenleitner 2013: 27; Fateryga 2017: 182 (as synonym of E.mediterraneus).
Eumenes (Eumenes) mediterraneus bengasinus ; van der Vecht and Fischer 1972: 129; Dal Pos et al. 2022: 15.
Eumenes mediterraneus cypricus Blüthgen, 1938: 488; Gusenleitner 2013: 27; Fateryga 2017: 182 (as synonym of E.mediterraneus).
Eumenes (Eumenes) mediterraneus cypricus ; van der Vecht and Fischer 1972: 129; Dal Pos et al. 2022: 15.
Eumenes (Eumenes) houskai Giordani Soika, 1952a: 17; van der Vecht and Fischer 1972: 128; Fateryga 2017: 182 (as synonym of E.mediterraneus).
Eumenes (Eumenes) mediterraneus anatolicus Giordani Soika, 1952b: 376; van der Vecht and Fischer 1972: 129; Fateryga 2017: 182 (as synonym of E.mediterraneus).
Eumenes mediterraneus manchurianus Giordani Soika, 1971: 70; Gusenleitner 1999: 572; Fateryga 2017: 182 (as synonym of E.mediterraneus).
Eumenes (Eumenes) mediterraneus manchurianus ; Dal Pos et al. 2022: 15.
Eumenes mediterraneus var. opacus Gusenleitner, 1972: 92; Fateryga 2017: 182 (as synonym of E.mediterraneus).
Eumenes mediterraneus filitosa Gereys, 2011: 224–225, 2016: 132; Frommer 2012: 179.

##### Notes.

**Figures 94–102.**
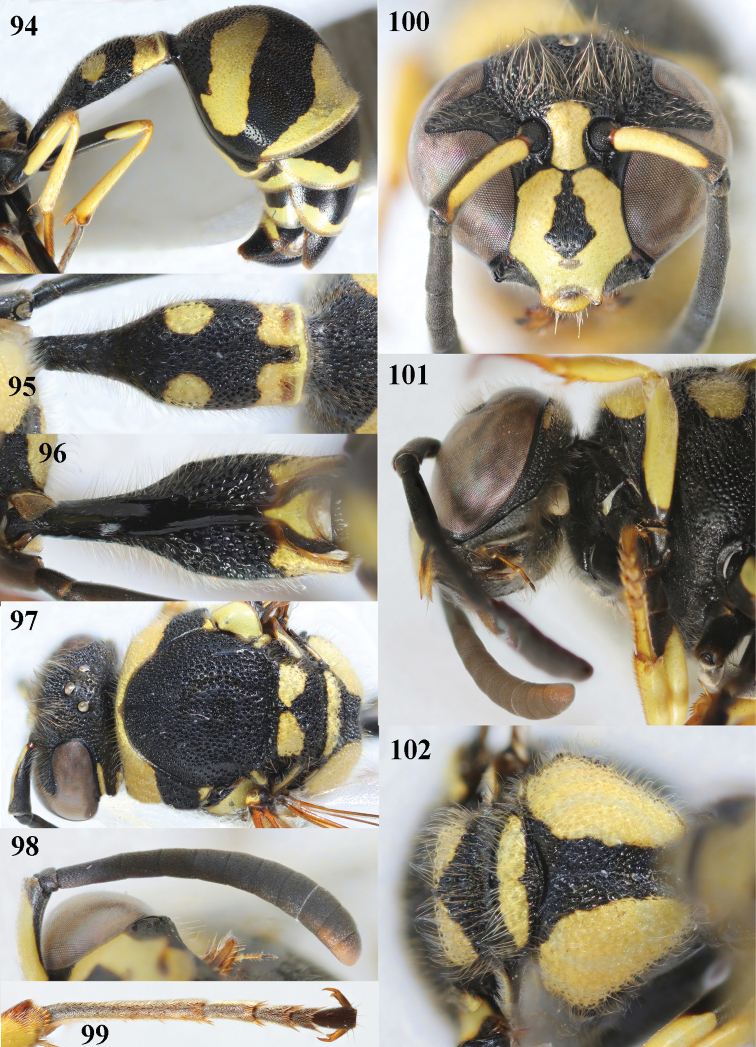
*Eumenesmediterraneus* Kriechbaumer, Bulgaria, female **94** metasoma lateral **95** first metasomal tergite dorsal **96** first tergite ventral **97** mesosoma dorsal **98** antenna anterior **99** hind tarsus and tarsal claws **100** head anterior **101** head and propleuron lateral **102** propodeum dorsal.

This species is in need of a critical revision; the few molecular data indicate that several cryptic species may be included under *E.mediterraneus* (Fig. 3). The lectotype male of *E.mediterraneus* originates from Croatia (Dalmatia) and was examined digitally by photographs kindly supplied by Stephan and Olga Schmidt (ZSM). It has the apical hook of the antenna less curved than pictured in Fig. 111 and its basal half densely setose. The sampled specimens from Crete and Corsica are different (Fig. 3) and a large-scale revision with sufficient fresh material from all over Europe is needed to sort out the relationships within the *E.mediterraneus* complex. For the populations of Corsica and Sardinia the name of *E.m.filitosa* Gereys is available; supposed to differ in most cases by the entirely black fifth tergite or largely so because of one or more small yellow patch(es) (in *E.mediterraneus* usually with complete yellow apical band, but absent in figured typical *E.mediterraneus* (Fig. 94)). Possibly the strongly convex second metasomal tergite and deeper subposterior depression may be of importance for its separation. For the population of Cyprus ssp. cypricus Blüthgen is available and differs by having the punctures of vertex, mesoscutum and second metasomal tergite at least twice larger than in typical *E.mediterraneus* (Gusenleitner 1972).

**Figures 103–111.**
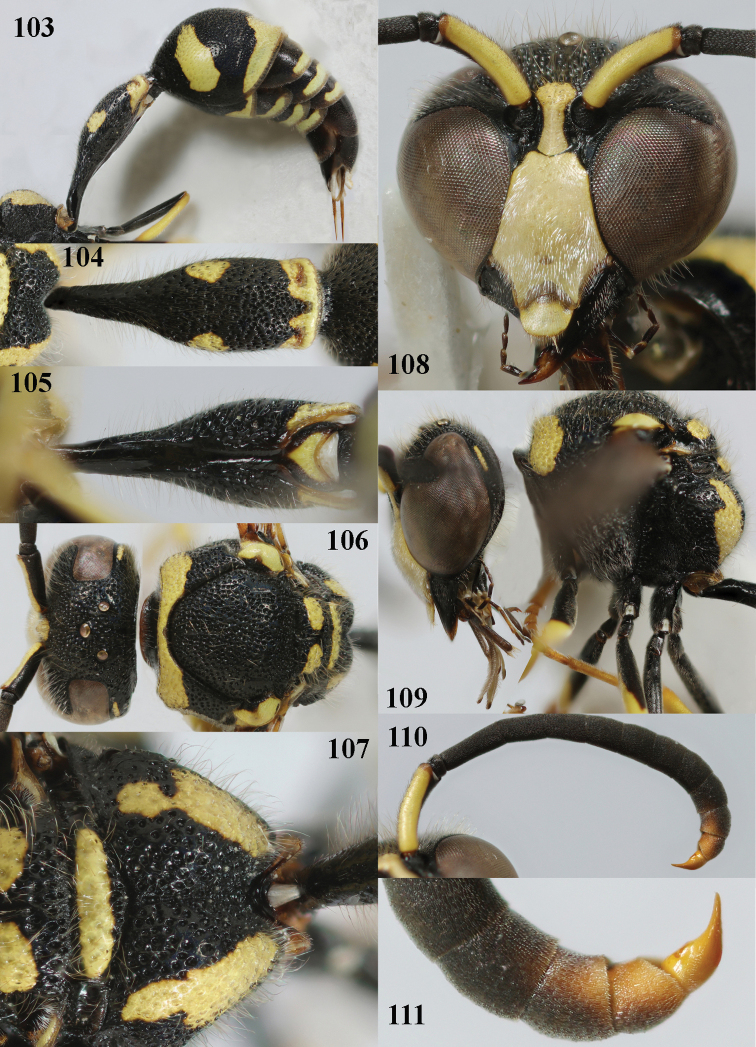
*Eumenesmediterraneus* Kriechbaumer, Bulgaria, male **103** metasoma lateral **104** first metasomal tergite dorsal **105** first tergite ventral **106** head and mesosoma dorsal **107** propodeum dorsal **108** head anterior **109** head and mesosoma lateral **110** antenna anterior **111** apical hook of antenna lateral.

##### Distribution.

Mediterranean, Balkan Peninsula, rarely in Central Europe (e.g., Switzerland only in Ticino and Valais and late in season (July–October; Neumeyer 2019) and very rarely collected in Germany (Frommer 2012; Reder 2022). In Greece starting in April and present in lowland and submontane habitats (Arens 2012). Reported from Asia up to Turkey, Iran, Afghanistan, Saudi-Arabia, China, Korea, and India, but this probably will change after a full revision (including molecular research) considering the uncertainty about the number of taxa under *E.mediterraneus* in Europe.

#### ﻿ Eumenes papillarius

Taxon classificationAnimaliaHymenopteraVespidae

(Christ, 1791)

198D3A48-865D-59F4-B6DD-6695854AD86B

Figs 112–121 , 122–131 , 262–266

Sphex papillarius Christ, 1791: 325.
Eumenes papillarius ; Tobias and Kurzenko 1978: 160; Hensen 1985: 45; Castro 1997: 4; Schmid-Egger and Schmidt 2002: 18; Schmid-Egger 2004: 72, 2010: 23; Smit et al. 2004: 327; Woydak 2006: 41–44; Castro and Sanza 2009: 266; Abenius 2012: 272; Arens 2012: 488; Archer 2014: 33; Neumeyer 2014: 367, 2019: 272; Baldock et al. 2020: 44.
Eumenes (Eumenes) papillarius papillarius ; van der Vecht and Fischer 1972: 130 (literature before 1972); Vergés Serra 1985: 143; Castro 1997: 4; Sanza 1997: 451; Gereys 2016: 132; Dal Pos et al. 2022: 16.
Eumenes papillarius papillarius ; Gusenleitner 1972: 87–88, 2013: 28; Borsato and Turrisi 2004: 144; Gogala 2022: 26–28.
Eumenes (Eumenes) papillarius ; Fateryga 2017: 182, 2018: 207.
Eumenes bipunctis de Saussure, 1852: 33; van der Vecht and Fischer 1972: 134 (as unidentified species; literature before 1972); Gusenleitner 1972: 87 (as synonym of E.papillarius); Gereys 2016: 132 (id.); Fateryga 2017: 182 (id.).
Eumenes bimaculatus André, 1884: 645; van der Vecht and Fischer 1972: 130 (as synonym of E.papillarius; literature before 1972): Gusenleitner 1972: 87 (as synonym of E.papillarius); Gereys 2016: 132 (id.); Fateryga 2017: 182 (id.).
Eumenes papillarius var. baltica Blüthgen, 1938: 485; Fateryga 2017: 182 (as synonym of E.papillarius).
Eumenes (Eumenes) papillarius balticus ; van der Vecht and Fischer 1972: 130 (literature before 1972); Dal Pos et al. 2022: 16.
Eumenes papillarius balticus ; Gusenleitner 1972: 89–90, 1999: 572.
Eumenes mediterraneus aemilianus Guiglia, 1951: 28. Syn. nov.
Eumenes (Eumenes) mediterraneus aemilianus ; van der Vecht and Fischer 1972: 125 (literature before 1972).
Eumenes aemilianus ; Gusenleitner 1972: 95–96, 1999: 569; Borsato 2006: 140.
Eumenes papillarius monticola Blüthgen, 1956: 2; van der Vecht and Fischer 1972: 130; Castro 1997: 4 (as synonym of E.papillarius); Gereys 2016: 132 (id.); Fateryga 2017: 182 (id.).
Eumenes papillarius rubricornis Giordani Soika (in Gusenleitner), 1972: 90; Gusenleitner 1999: 572, 2013: 28; Fateryga 2017: 182 (as synonym of E.papillarius).
Eumenes (Eumenes) papillarius rubricornis ; Dal Pos et al. 2022: 16.

##### Notes.

Large specimens (fore wing length about 10 mm) have frequently a pair of yellow patches on the mesoscutum antero-laterally; the patches vary from minute to large. The photographs of the female holotype kindly supplied by Roberto Poggi (MSNG) show that *E.aemilianus* Guiglia, 1951 is a junior synonym of *E.papillarius* (Christ, 1791) (syn. nov.) because of the entirely dark brown labrum (Fig. 265), the shallow apical emargination of the clypeus (Fig. 265), the pair of large yellow patches on the mesoscutum (Fig. 264) and the medium-sized setosity of the second tergite (mentioned in the original description). The holotype female (deposited in MSNG) is a comparatively yellowish specimen with slender first tergite, it has the clypeus narrowly black apically, the mesoscutum with a pair of large and curved yellow lateral patches, the apex of the antenna black and the apical rim of the second tergite pale yellowish (Figs 262–266). The presence or absence of a pair of yellow mesoscutal patches is a variable (and at least partly size related) character in *E.papillarius* and does not indicate that it concerns a separate species (see also comments of Gusenleitner (1972) under *E.papillarius*). Aberrant specimens from the Iberian Peninsula have medium-sized to long setae on the second sternite and the apex of the hind tibia is yellow. The apical lamella of the second tergite varies from pale yellowish to dark brown.

**Figures 112–121.**
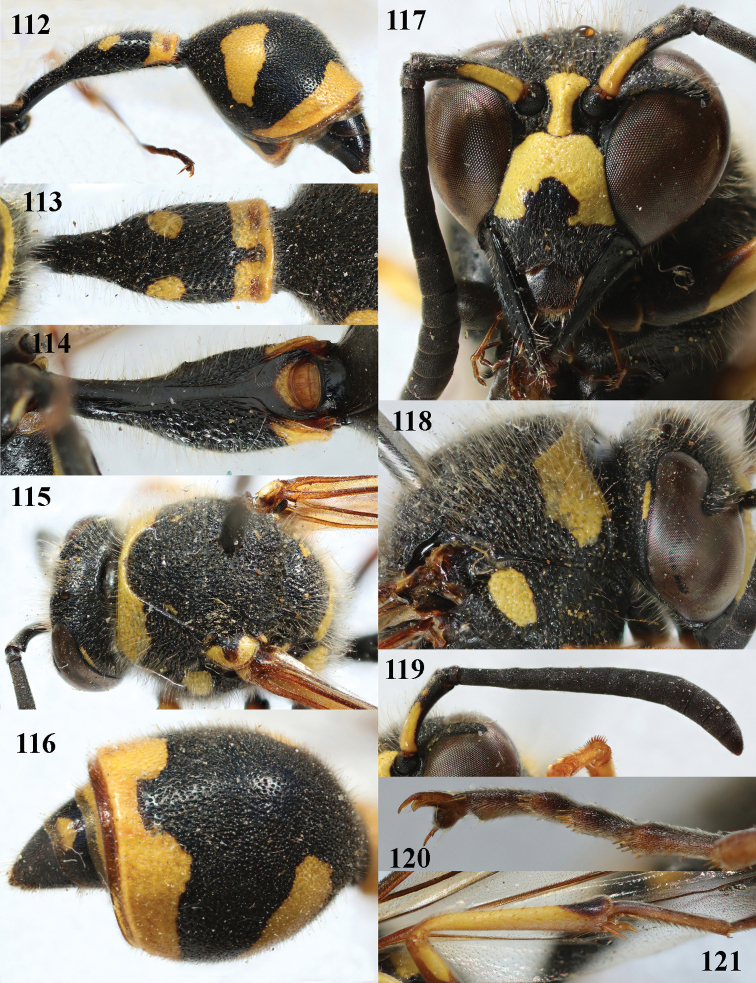
*Eumenespapillarius* (Christ), France, female **112** metasoma lateral **113** first metasomal tergite dorsal **114** first tergite ventral **115** head and mesosoma dorsal **116** second tergite latero-dorsal **117** head anterior **118** head and pronotum lateral **119** antenna anterior **120** hind tarsal claw **121** hind tibia lateral.

**Figures 122–131.**
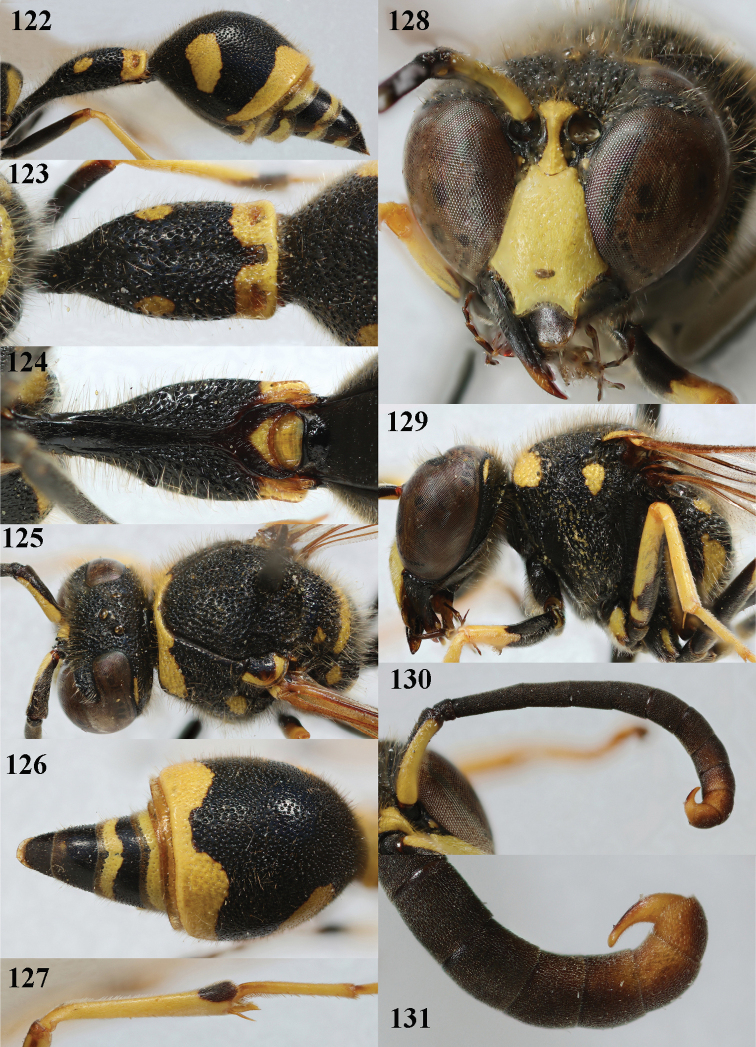
*Eumenespapillarius* (Christ), France, male **122** metasoma lateral **123** first metasomal tergite dorsal **124** first tergite ventral **125** head and mesosoma dorsal **126** second tergite dorsal **127** hind tibia lateral **128** head anterior **129** head and mesosoma lateral **130** antenna anterior **131** apical hook of antenna lateral.

##### Distribution.

Widespread in most of Europe, but considered absent from UK (only known as vagrant in England; Archer 2014), Norway, Sweden and Finland. Found up to 1550 m altitude in Switzerland (Neumeyer 2019), but in S Greece rather rare and restricted to lowland and submontane habitats (Arens 2012). This species is often found near human settlements and using small crevices of buildings (e.g., under roof tiles) to construct small groups of clay nests.

#### ﻿ Eumenes pedunculatus

Taxon classificationAnimaliaHymenopteraVespidae

(Panzer, 1799)

E93F8892-F62E-57CC-83F9-1BF9DDAD1F7A

Figs 132–139 , 140–149

Vespa pedunculata Panzer, 1799: (6) 63: 8, pl. 8.
Eumenes (Eumenes) pedunculatus pedunculatus ; van der Vecht and Fischer 1972: 131 (literature before 1972); Vergés Serra 1985: 148; Castro 1997: 4; Sanza 1997: 455; Schmid-Egger 2004: 72; Frommer 2013: 19; Gereys 2016: 135; Dal Pos et al. 2022: 16.
Eumenes pedunculatus ; Tobias and Kurzenko 1978: 160; Hensen 1985: 45; Schmid-Egger and Schmidt 2002: 18; Smit et al. 2004: 327; Woydak 2006: 44–46; Castro and Sanza 2009: 266; Schmid-Egger 2010: 23, 2011: 44; Abenius 2012: 273; Arens 2012: 487–488; Neumeyer 2014: 367, 2019: 273; Fateryga et al. 2020: 96; Baldock et al. 2020: 44.
Eumenes pedunculatus pedunculatus ; Gusenleitner 1972: 81–82; Gogala 2022: 29–30.
? Eumenesmarginella Herrich-Schäffer, 1841: 44, pl. 179-8.
? Eumenes (Eumenes) marginellus; van der Vecht and Fischer 1972: 134 (as unidentified species; literature before 1972).
Eumenes obscurus André, 1884: 636–637. Syn. nov.
Eumenes andrei Dalla Torre, 1894: 17 (new name for junior homonym E.obscurus André); van der Vecht and Fischer 1972: 134 (literature before 1972). Syn. nov.
Eumenes eburneopictus Giordani Soika, 1940: 97; Fateryga 2017: 182 (as synonym of E.pedunculatus).
Eumenes (Eumenes) eburneopictus ; van der Vecht and Fischer 1972: 127.
Eumenes pedunculatus eburneopictus ; Gusenleitner 1999: 572.
Eumenes pedunculatus turanus Blüthgen, 1943: 302; Gusenleitner 1972: 82, 1999: 573; Fateryga 2017: 182 (as synonym of E.pedunculatus).
Eumenes (Eumenes) pedunculatus turanus ; van der Vecht and Fischer 1972: 131; Dal Pos et al. 2022: 16.
Eumenes pedunculata var. lapponica Hellén, 1944: 11; van der Vecht and Fischer 1972: 131 (as synonym of E.pedunculatus); Gusenleitner 1972: 81 (id.); Gereys 2016: 135 (id.); Fateryga 2017: 182 (id.).
Eumenes karafutonis Yamane, 1977: 61–62; Fateryga 2017: 182 (as synonym of E.pedunculatus).

##### Notes.

The depository of the female holotype of *E.obscurus* André (= *E.andrei* Dalla Torre) is unknown, but the extensive description allows identification. The robust posterior part of the first tergite (in dorsal view campaniform), the entirely dark antenna, the black clypeus except for a yellow dorsal linear patch and the shiny and very finely punctate second tergite points to *E.pedunculatus* (Panzer). The type series of *E.marginellus* is lost; the more or less yellow scape, the black scutellum, the narrow yellow patch of the pronotum and narrow yellow posterior patch of the first tergite are similar to some examined specimens of *E.pedunculatus*.

##### Distribution.

Widely distributed in Europe but relatively rare in collections from NW and S Europe (e.g., only *Eumenes* sp. known from Norway, absent from UK and Corsica, in S Europe rare and restricted to montane habitats (Arens 2012; Gereys 2016)). Outside Europe known from the East Palaearctic region up to Japan and Korea. Associated with *Calluna-Pinus* heaths (Woydak 2006) and occurring up to 1850 m altitude in Switzerland (Neumeyer 2019) and 2550 m in Spain (Gereys 2016).

**Figures 132–139.**
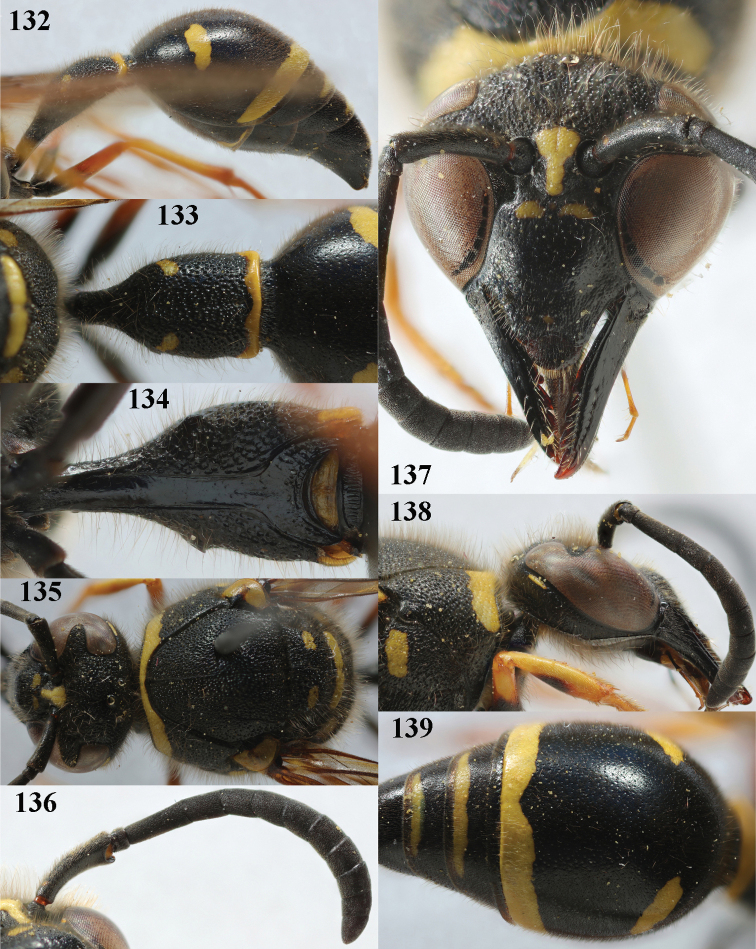
*Eumenespedunculatus* (Panzer), Netherlands (Witteveen), female **132** metasoma lateral **133** first metasomal tergite dorsal **134** first tergite ventral **135** head and mesosoma dorsal **136** antenna anterior **137** head anterior **138** head and pronotum lateral **139** second tergite latero-dorsal.

**Figures 140–149.**
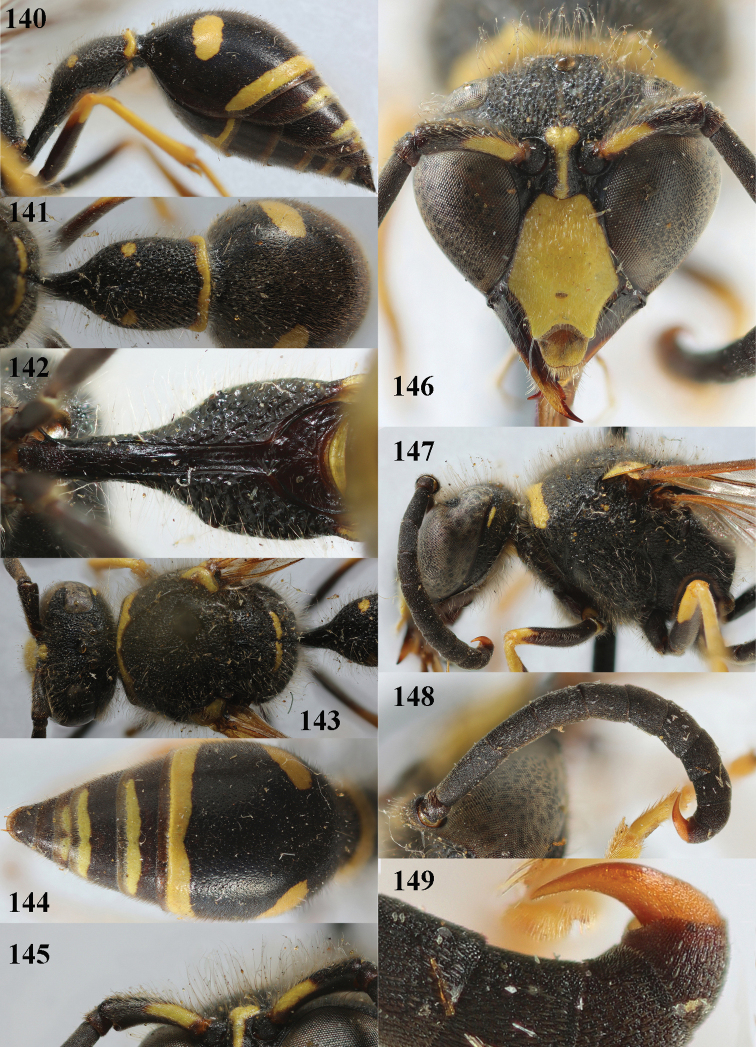
*Eumenespedunculatus* (Panzer), Netherlands (Helenaveen), male **140** metasoma lateral **141** first metasomal tergite dorsal **142** first tergite ventral **143** head and mesosoma dorsal **144** second tergite dorsal **145** scape anterior **146** head anterior **147** head and mesosoma lateral **148** antenna anterior **149** apical hook of antenna lateral.

#### ﻿ Eumenes pomiformis

Taxon classificationAnimaliaHymenopteraVespidae

(Fabricius, 1781)

8DC22D50-A346-5E10-B95F-89506C2A92E2

Figs 1b , 150–158 , 159–169

Vespa pomiformis Fabricius, 1781: 467.
Eumenes pomiformis pomiformis ; Gusenleitner 1972: 99–100, 1997: 144, 1999: 573; Borsato and Turrisi 2004: 145.
Eumenes pomiformis ; Tobias and Kurzenko 1978: 160; Vergés Serra 1985: 147; Giordani Soika and Borsato 1995: 7; Sanza 1997: 458; Castro 1997: 4; Schmid-Egger and Schmidt 2002: 18; Schmid-Egger 2004: 73, 2010: 23; Borsato 2006: 143; Castro and Sanza 2009: 266; Arens 2012: 486–487; Gusenleitner 2013: 28; Neumeyer 2014: 367, 2019: 269; Baldock et al. 2020: 44; Gogala 2022: 29–30; Cassar et al. 2022: 207–208.
Eumenes (Eumenes) pomiformis ; van der Vecht and Fischer 1972: 131–132 (literature before 1972); Castro 1992: 25; Gereys 2016: 135–136; Fateryga 2017: 182, 2018: 207–208; Dal Pos et al. 2022: 16.
? Vespahistrio de Villers, 1789: 282–283. Type series lost.
? Eumenes (Eumenes) histrio; van der Vecht and Fischer 1972: 134 (as unidentified species).
Eumenis (sic!) mediterraneavar.heri Kriechbaumer, 1879: 88; van der Vecht and Fischer 1972: 131 (as synonym of E.pomiformis); Gereys 2016: 135 (id.); Fateryga 2017: 182 (id.).
Eumenes fastidiosissimus Giordani Soika, 1943: 29; van der Vecht and Fischer 1972: 131 (as synonym of E.pomiformis); Gusenleitner 1972: 99 (id.); Gereys 2016: 135 (id.); Fateryga 2017: 182 (id.).
Eumenes pomiformis turcicus Giordani Soika, 1952: 367; van der Vecht and Fischer 1972: 132 (holotype, but part of paratypes belong to E.lunulatus); Gusenleitner 1972: 101, 2013: 28 (as synonym of E.pomiformis); Gereys 2016: 135 (id.); Fateryga 2017: 182 (id.).

##### Notes.

The female lectotype of *E.pomiformis* was examined digitally by photographs kindly supplied by Sree Gayathree Selvantharan and Lars Vilhelmsen (NHMD), as the male holotype of *E.heri* (photographs kindly supplied by Stephan and Olga Schmidt (ZSM)). The latter has the apical hook of the antenna less flattened than figured in Fig. 169. Unfortunately, the male lectotype of *E.fastidiossimus* deposited in Museo Civico di Storia Naturale, Venezia (see Giordani Soika 1973) could not be found. Traditionally, *E.fastidiosissimus* is synonymised with *E.pomiformis* (e.g., Gusenleitner 1972; van der Vecht and Fischer 1972) which is not contradicted by the (incomplete) original description.

##### Distribution.

One of the common species in S Europe, reaching Germany (but very rarely collected) and Belarus. Known from Corsica, Sardinia, Sicily, and Malta (Gereys 2016; Cassar et al. 2022). In southern Switzerland this fairly common species occurs up to 1600 m (Neumeyer 2019) and up to 1900 m in Greece (Arens 2012). Outside Europe known from Tunisia, Lebanon, Turkey, and China. The report from India (Kumar et al. 2017) is doubtful because of the differences in the shape of the first tergite.

**Figures 150–158.**
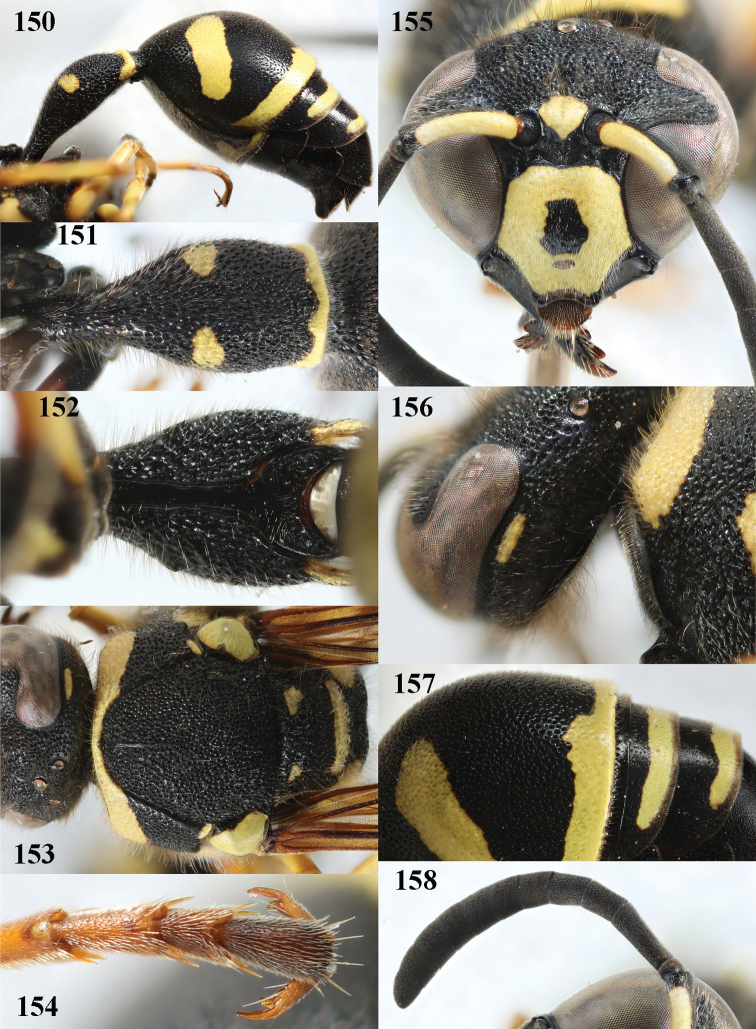
*Eumenespomiformis* (Fabricius), Bulgaria, female **150** metasoma lateral **151** first metasomal tergite dorsal **152** first tergite ventral **153** mesosoma dorsal **154** hind tarsal claws **155** head anterior **156** head and pronotum lateral **157** second tergite latero-dorsal **158** antenna anterior.

**Figures 159–169.**
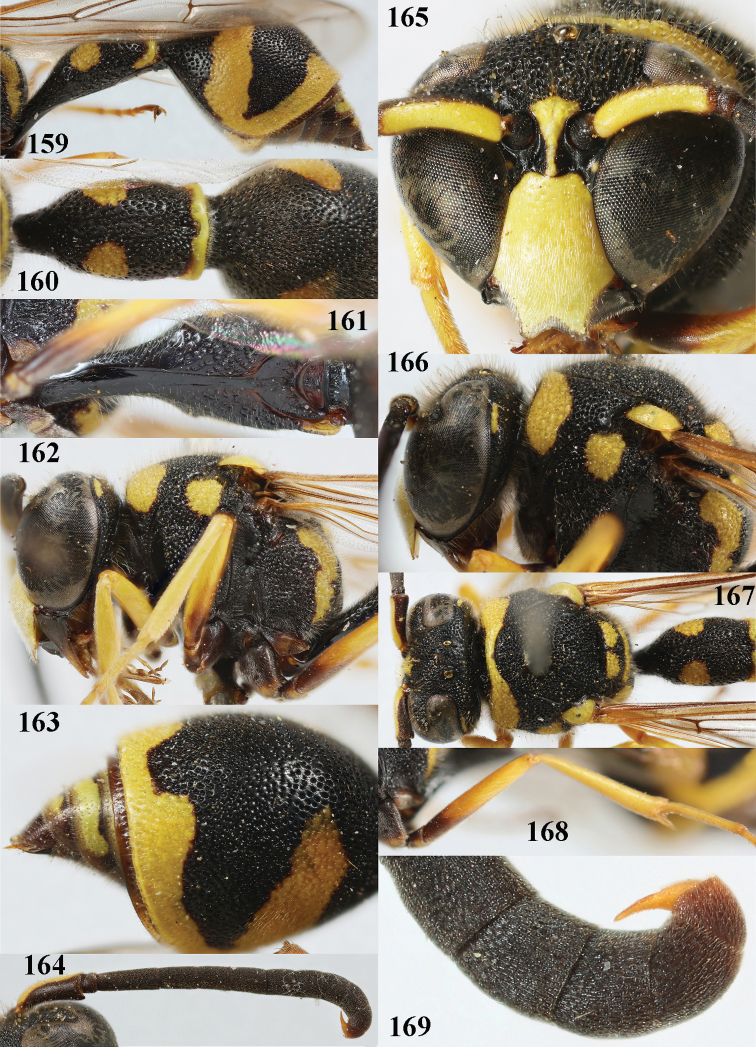
*Eumenespomiformis* (Fabricius), Italy, male **159** metasoma lateral **160** first metasomal tergite dorsal **161** first tergite ventral **162** head and mesosoma lateral **163** second tergite dorso-lateral **164** antenna **165** head anterior **166** head and mesosoma lateral **167** head and mesosoma dorsal **168** hind femur and tibia lateral **169** apical hook of antenna lateral.

#### ﻿ Eumenes punctaticlypeus

Taxon classificationAnimaliaHymenopteraVespidae

Giordani Soika, 1943

644D24DB-7A8A-574C-80A7-16BD4FD9BBE7

Figs 170–179 , 180–188

Eumenes robusta Kostylev, 1940: 141 (primary homonym; not E.robustus Isely, 1917).
Eumenes (Eumenes) robustus ; van der Vecht and Fischer 1972: 132.
Eumenes (Eumenes) punctaticlypeus Giordani Soika, 1943: 29; van der Vecht and Fischer 1972: 132 (literature before 1972); Gusenleitner 1972: 104–105; Gereys 2016: 137; Fateryga 2017: 182, 2018: 208; Dal Pos et al. 2022: 16.
Eumenes punctaticlypeus punctaticlypeus ; Gusenleitner, 1999: 573.
Eumenes punctaticlypeus ; Castro 1997: 4; Castro and Sanza 2009: 266–267; Arens 2012: 488–489; Baldock et al. 2020: 44.
Eumenes (Eumenes) calabricus Giordani Soika, 1943: 31; Giordani Soika 1956: 316; van der Vecht and Fischer 1972: 132; Gusenleitner 1972: 104–105 (as synonym of E.punctaticlypeus); Gereys 2016: 137 (id.).
Eumenes kostylevi Kurzenko, 1976: 437 (replacement name for E.robusta); Tobias and Kurzenko 1978: 161; Gereys 2016: 137 (as synonym of E.punctaticlypeus).
Eumenes kostylevi kostylevi ; Yildirim and Özbek 1996: 206.
Eumenes kostylevi punctaticlypeus ; Yildirim and Özbek 1996: 206.
Eumenes punctaticlypeus kostylevi ; Fateryga and Matushkina 2010: 377 (biology); Fateryga 2010: 78.

##### Notes.

As shown by the short setae of the hind coxa, the robust posterior part of the first metasomal tergite and frequently present moon-shaped yellow patch of the clypeus in females of both *E.punctaticlypeus* and *E.lunulatus*, the first one could be considered a large form of the latter. We recognise *E.punctaticlypeus* as a separate species because of the dark brown antennal hook of the males (yellow in *E.lunulatus*), differences in sculpture (but part may be the result of the larger body size) and the presence of a pair of large yellow spots on the mesoscutum of females (but the latter is variable in *E.papillarius* and this may be also the case in this species).

##### Distribution.

Examined specimens are from Spain, France, Bulgaria, and Turkey. This rarely collected species is also reported from Albania, Italy (type series), Greece and Ukraine (Crimea).

#### ﻿ Eumenes sardous

Taxon classificationAnimaliaHymenopteraVespidae

Guiglia, 1951

4ED7623D-B4A2-5940-A215-146A510FDBD2

Figs 189–197 , 198–207

Eumenes sardous Guiglia, 1951: 27; Gusenleitner 1972: 108, 1999: 573; Borsato 2006: 143.
Eumenes (Eumenes) sardous ; van der Vecht and Fischer 1972: 133 (literature before 1972); Gereys 2016: 137; Dal Pos et al. 2022: 16.

##### Notes.

Similar to *E.subpomiformis* according to Blüthgen (1954) and Gusenleitner (1972), which especially counts for the males. We refrain from synonymising this species till we have more data (COI, biology), also because Sardinia and Corsica are known to have a relative high degree of endemicity.

**Figures 170–179.**
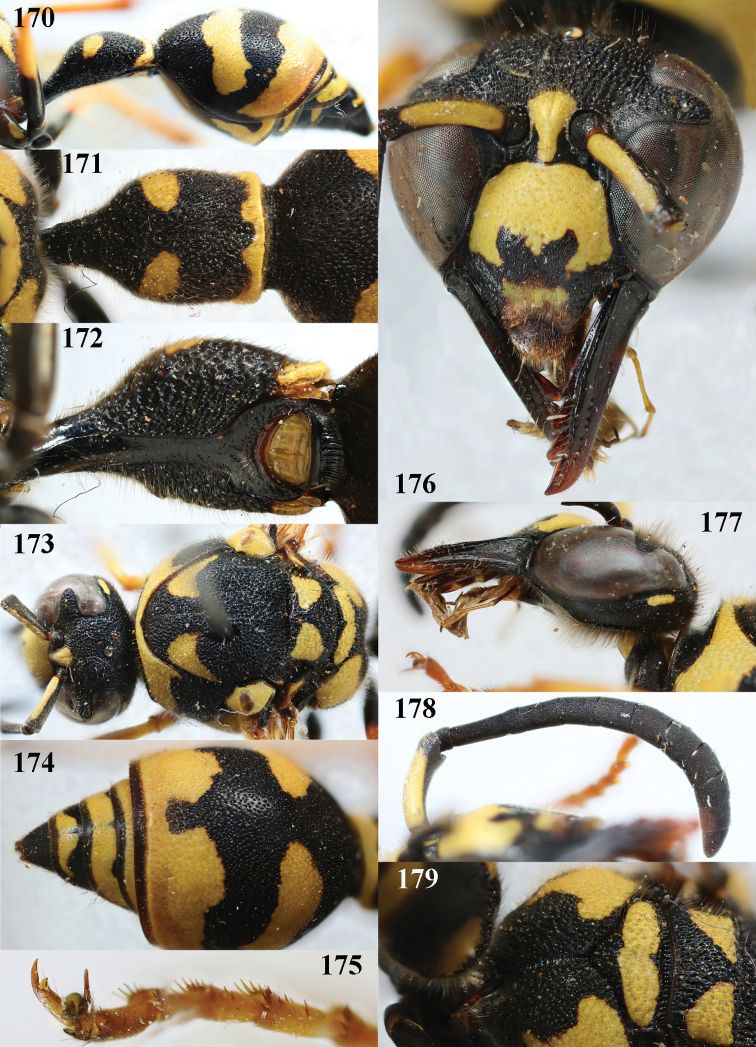
*Eumenespunctaticlypeus* Giordani Soika, France, female **170** metasoma lateral **171** first metasomal tergite dorsal **172** first tergite latero-ventral **173** head and mesosoma dorsal **174** second tergite latero-dorsal **175** hind tarsal claw **176** head anterior **177** head and pronotum lateral **178** antenna anterior **179** propodeum dorsal.

**Figures 180–188.**
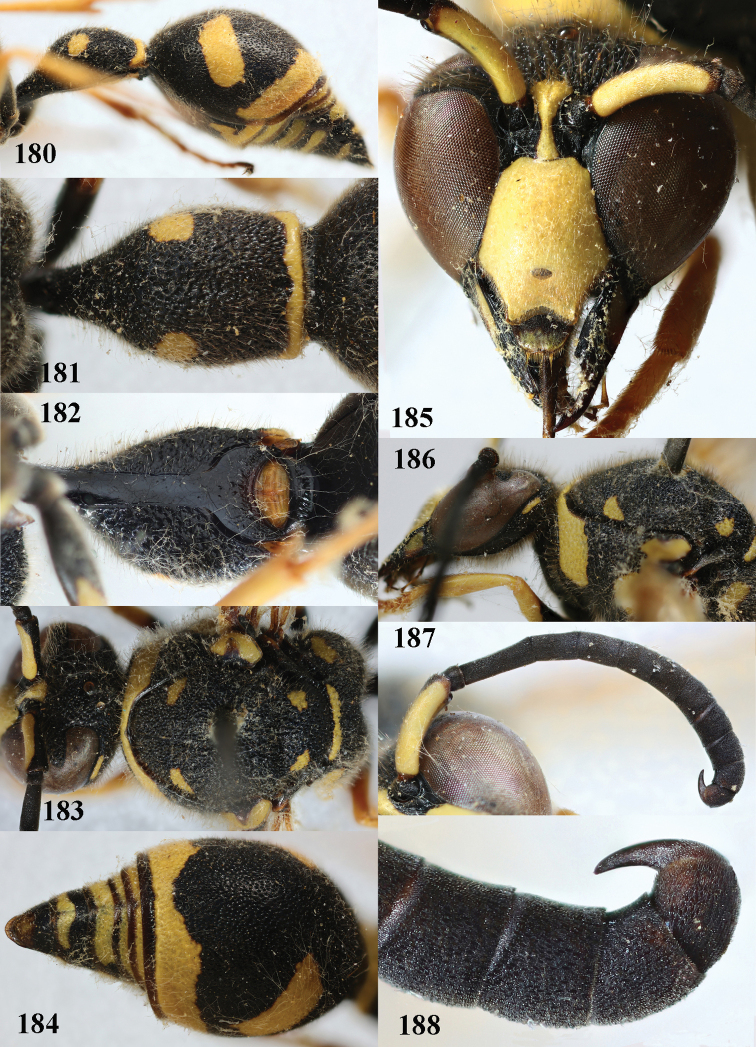
*Eumenespunctaticlypeus* Giordani Soika, Spain, male **180** metasoma lateral **181** first metasomal tergite dorsal **182** first tergite ventral **183** head and mesosoma dorsal **184** second tergite dorso-lateral **185** antenna **186** head anterior **187** antenna anterior **188** apical hook of antenna lateral.

**Figures 189–197.**
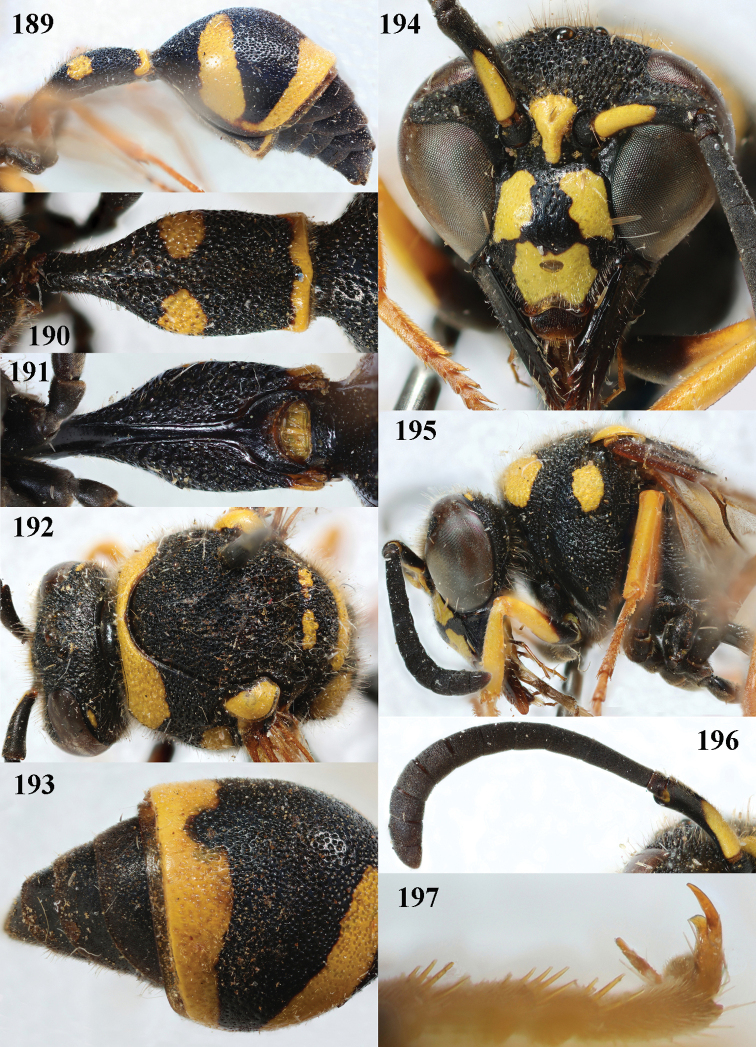
*Eumenessardous* Guiglia, France (Corsica), female **189** metasoma lateral **190** first metasomal tergite dorsal **191** first tergite ventral **192** head and mesosoma dorsal **193** second tergite latero-dorsal **194** head anterior **195** head and mesosoma lateral **196** antenna anterior **197** hind tarsal claw.

**Figures 198–207.**
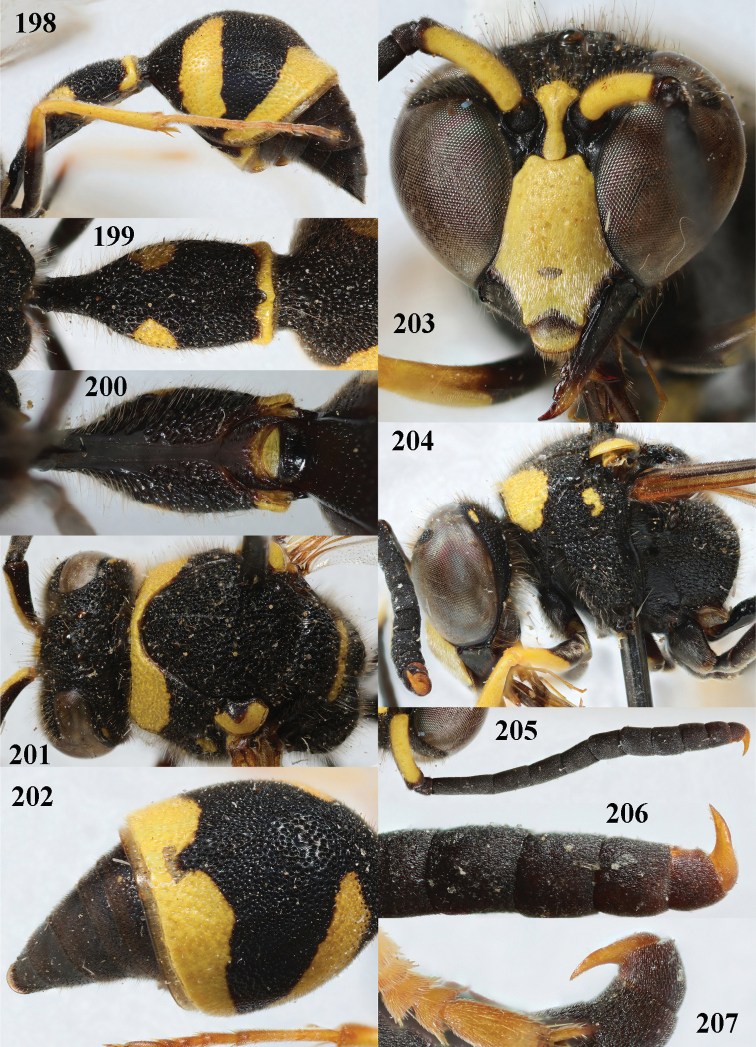
*Eumenessardous* Guiglia, France (Corsica), male **198** metasoma lateral **199** first metasomal tergite dorsal **200** first tergite ventral **201** head and mesosoma dorsal **202** second tergite dorso-lateral **203** head anterior **204** head and mesosoma lateral **205** antenna anterior **206** apical hook of antenna lateral **207** id. of other antenna.

##### Distribution.

An endemic species of Sardinia (Italy) and Corsica (France), occurring from sea level up to 1600 m altitude in Corsica (Gereys 2016).

#### ﻿ Eumenes sareptanus

Taxon classificationAnimaliaHymenopteraVespidae

André, 1884

369B75A3-D695-5B04-9D54-376892DC8B42

Figs 208–215 , 216–226

Eumenes sareptanus André, 1884: 638; Tobias and Kurzenko 1978: 160; Arens 2012: 486; Neumeyer 2014: 367, 2019: 275; Cassar et al. 2022: 208.
Eumenes (Eumenes) sareptanus sareptanus ; van der Vecht and Fischer 1972: 133 (literature before 1972).
Eumenes sareptanus sareptanus ; Gusenleitner 1972: 108–109, 2013: 28; Dal Pos et al. 2022: 16.
Eumenes (Eumenes) sareptanus ; Fateryga 2017: 182, 2018: 208.
Eumenes pomiformis f. insolata Müller, 1923: 627; Fateryga 2017: 182 (as synonym of E.sareptanus).
Eumenes (Eumenes) sareptanus insolatus ; van der Vecht and Fischer 1972: 133 (literature before 1972); Castro 1992: 25, 30 (as synonym of E.dubius).
Eumenes sareptanus insolatus ; Gusenleitner 1972: 180; Schmid-Egger and Schmidt 2002: 18; Schmid-Egger 2010: 23.
Eumenes dubius sareptanus var. germanica Blüthgen, 1938: 469, 474, 495; van der Vecht and Fischer 1972: 133 (as synonym of E.sareptanusinsolatus; literature before 1972); Fateryga 2017: 182 (as synonym of E.sareptanus).
Eumenes dubius crimensis Blüthgen, 1938: 468–469; van der Vecht and Fischer 1972: 127.
Eumenes (Eumenes) crimensis ; Gusenleitner 2013: 27; Fateryga 2017: 181, 2018: 205–206; Dal Pos et al. 2022: 15.
Eumenes sareptanus scabrosus ; Fateryga 2017: 181 (as synonym of E.crimensis).

##### Notes.

Castro (1992) concluded that traditional characters do not suffice to separate *E.sareptanus* from *E.dubius* after studying a large series of Spanish specimens. The observed variation is considered to be clinal and connected to climatic conditions (indicated by latitude and altitude). Therefore, both Castro (1992) and Gereys (2016) consider all European specimens of *E.sareptanus* and *E.dubius* conspecific, with the latter as oldest and thus valid name. It is obvious that with the differences used in existing keys both species are not well separable, this is especially the case for the females. The apical antennal hook of *E.sareptanus* is generally more slender than in *E.dubius* (Figs 224, 226*versus* Figs 89, 92), albeit that the difference is less obvious in part of the SW European males. Traditionally, *E.sareptanus* is separated from *E.dubius* by having the setae of the mesoscutum about as long as apical width of scape and the apical lamella of the second tergite longer than height of the preapical vertical depression of the tergite. In this paper we propose a different combination of characters, but it is obvious (also from the molecular data presented in Schmid-Egger and Schmidt (2021)) that both are valid species. More research is needed to clear up the interrelations in the group of *E.dubius* (viz., *E.dubius*, *sareptanus*, *cyrenaicus*).

**Figures 208–215.**
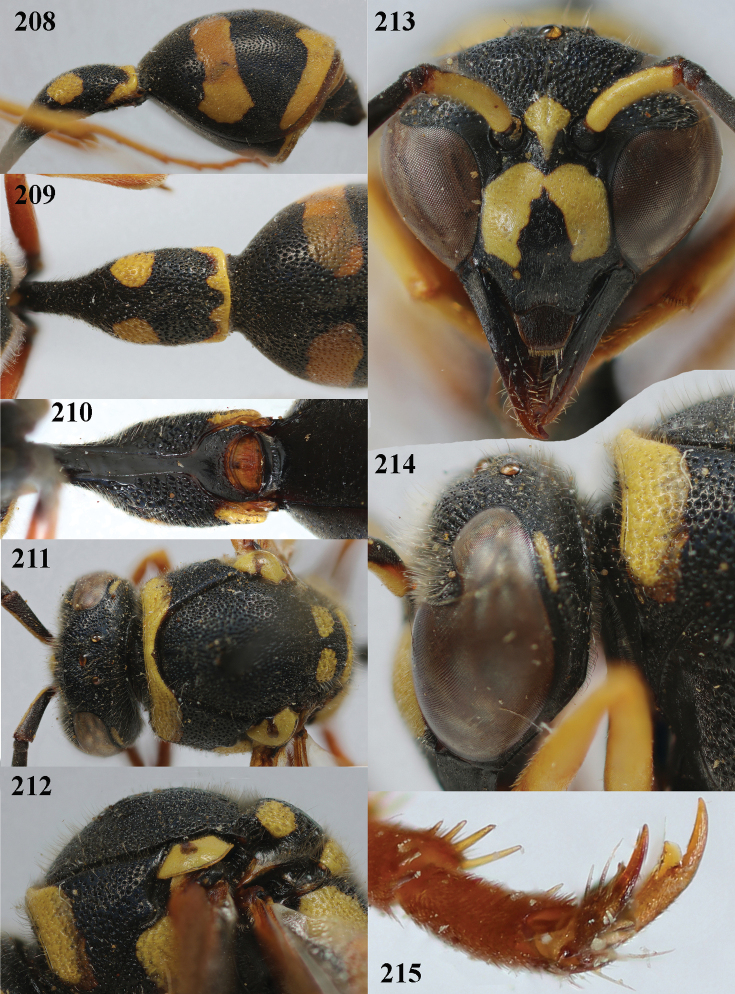
*Eumenessareptanus* André, Croatia, female **208** metasoma lateral **209** first metasomal tergite dorsal **210** first tergite ventral **211** head and mesosoma dorsal **212** mesoscutum and scutellum lateral **213** head anterior **214** head and propleuron lateral **215** hind tarsal claw.

**Figures 216–226.**
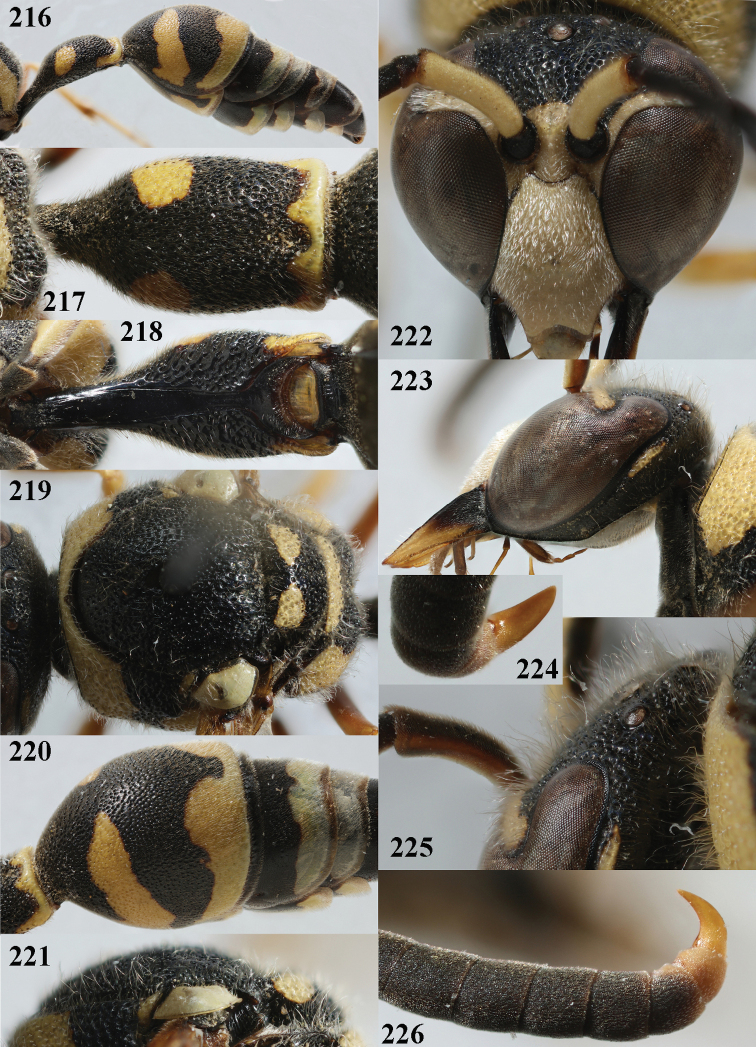
*Eumenessareptanus* André, Bulgaria, male **216** metasoma lateral **217** first metasomal tergite dorsal **218** first tergite ventral **219** mesosoma dorsal **220** second tergite latero-dorsal **221** mesoscutum and scutellum lateral **222** head anterior **223** head and mesosoma lateral **224** apical hook of antenna ventral **225** setosity of head latero-dorsal **226** antennal hook lateral.

Blüthgen (1938) described *Eumenesdubiuscrimensis* from Crimea (Jalta; only ♀-holotype and a ♂-paratype) mainly based on the coarser punctation of the mesoscutum (“Punktierung des Thorax grob, überwiegend met deutlichen, glatten, glänzenden Punktzwischenräumen, ….”) and length of its setae (intermediate between typical *E.sareptanus* (long setae) and typical *E.dubius* (short setae)). Gusenleitner (1972) examined a series from Crimea that contains only typical *E.dubius* specimens. In addition, he has seen specimens from Kopet Dag (= the border area of Turkmenistan and Iran) that fit better with the original description than specimens from Crimea. Fateryga (2018) agrees that these are different from the Crimean specimens and the specimens reported as *E.crimensis* from Iran, C Asia and Kazakhstan likely belong to *E.scabrosus* with slender aedeagi (Fateryga 2018). Gusenleitner (2013) treated *E.dubiuscrimensis* as a valid species without any comment, but the status of *E.crimensis* was discussed by Fateryga (2018). Considering the shape of the depicted aedeagi by Fateryga (2018) it could be *E.dubius* as well *E.sareptanus*; both have the medial part of the aedeagi similarly shaped.

Unfortunately, the original description does not include any remarks on the shape of the apical antennal segment of the male. The males should have the apical antennal segment narrower basally and less curved than in typical *E.dubius* according to Gusen­leitner (1972). Michael Greeff (ETHZ) kindly supplied the first author with photographs of the male paratype, which clearly shows that it is not *E.dubius* because of the comparatively slender apical antennal segment which fits well with the apical segment of *E.sareptanus*. The latter species has coarser punctures (often with distinct smooth interspaces) on head and mesosoma dorsally than in *E.dubius*, yellow area of pronotum more widened compared to median width, and first metasomal tergite latero-apically broadly yellow. All these characters are present in the paratype of *E.crimensis* and, therefore, we synonymise *E.crimensis* with *E.sareptanus* (syn. nov.).

##### Distribution.

A comparatively rarely collected species in C and S Europe as well in NW Asia. The typical form occurs in southern European Russia up to western Siberia. In Switzerland occurring between 255 and 1250 m altitude (Neumeyer 2019).

#### ﻿ Eumenes subpomiformis

Taxon classificationAnimaliaHymenopteraVespidae

Blüthgen, 1938

AACD0F70-42E2-572C-88D3-FCCEEA692360

Figs 227–235 , 236–243

Eumenes subpomiformis Blüthgen, 1938: 480, 496; Gusenleitner 1972: 101–103, 1999: 574, 2013: 29; Tobias and Kurzenko 1978: 161; Castro 1997: 4; Schmid-Egger and Schmidt 2002: 18; Woydak 2006: 46–47; Castro and Sanza 2009: 267; Arens 2012: 489; Schmid-Egger 2010: 23, 2011: 44; Neumeyer 2014: 367, 2019: 276; Baldock et al. 2020: 44.
Eumenes (Eumenes) subpomiformis ; van der Vecht and Fischer 1972: 133–134 (literature before 1972); Vergés Serra 1985: 147; Castro 1992: 25; Sanza 1997: 463; Schmid-Egger 2004: 73; Gereys 2006: 387, 2016: 137; Fateryga 2017: 182; Dal Pos et al. 2022: 16.
Eumenes subpomiformis subpomiformis ; Giordani Soika and Borsato 1995: 7; Borsato and Turrisi 2004: 145.
Eumenes subpomiformis crassipunctatus Blüthgen, 1956: 3; van der Vecht and Fischer 1972: 133 (literature before 1972); Gusenleitner 1972: 101–103 (as synonym of E.subpomiformis): Fateryga 2017: 182 (as synonym of E.sareptanus).

##### Notes.

As pointed out by Gusenleitner (1972)*E.subpomiformis* is very similar to *E.pomiformis* (“*Eumenespomiformis* steht der Art *subpomiformis* sehr nahe und nicht der Art *lunulatus*”) and is easily misidentified when the medium-sized or long setae of the propleuron of *E.subpomiformis* are not well exposed (head too much down), depressed or damaged. He also correctly denounced the differences in shape of the clypeus as illustrated by Blüthgen (1938) (“Die Form des Clypeus, wie sie Blüthgen für *subpomiformis* angibt (Ausschnittecken nach den Seiten gezogen) tritt auch bei *pomiformis* auf”.) What remains in both sexes for separation according to the keys by Gusenleitner (1972, 1999) is the length of the setae on the propleuron (with equal shorter setae in *E.pomiformis* and with unequal longer setae in *E.subpomiformis*). However, the setosity seems rather variable (especially in males) and should be used in combination with other characters. Recent molecular research (Neumeyer and Praz 2015; Schmid-Egger and Schmidt 2021; this paper) revealed distinct genetic differences between *E.subpomiformis* and *E.pomiformis* (Fig. 3) despite their overall similarity.

Specimens in RMNH identified by Blüthgen (in 1950 and 1955) as *E.pomiformisbarbatulus* belong either to *E.subpomiformis* (Portugal; females with mostly comparatively short setae on propleuron and deeply emarginate clypeus) or to *E.coarctatus* (most specimens (with medium-sized to long setae on propleuron) from Portugal, Spain, France, Algeria, Morocco).

**Figures 227–235.**
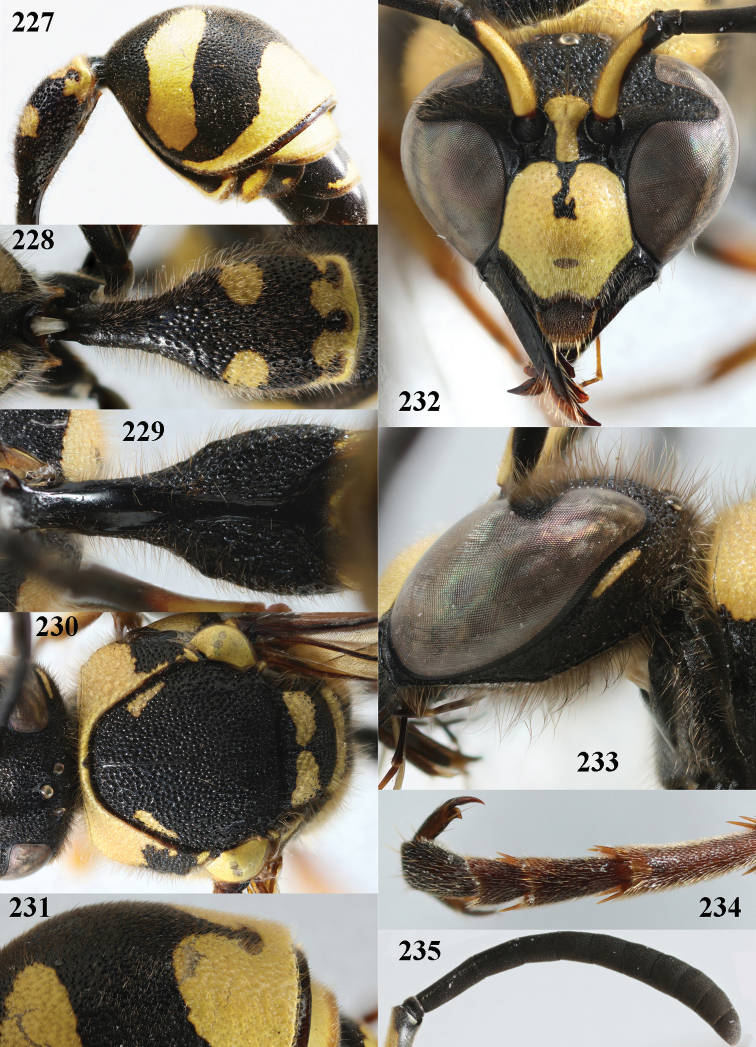
*Eumenessubpomiformis* Blüthgen, Bulgaria, female **227** metasoma lateral **228** first metasomal tergite dorsal **229** first tergite ventral **230** mesosoma dorsal **231** second metasomal tergite latero-dorsal **232** head anterior **233** head and propleuron lateral **234** hind tarsal claw **235** antenna.

**Figures 236–243.**
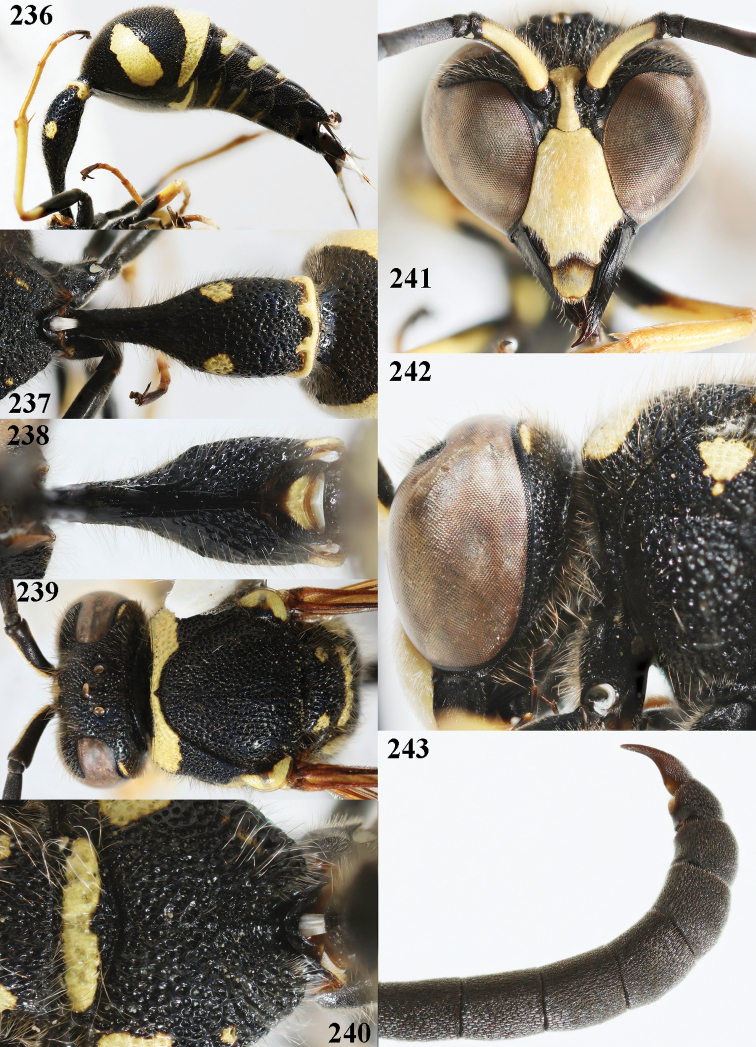
*Eumenessubpomiformis* Blüthgen, Bulgaria, male **236** metasoma lateral **237** first metasomal tergite dorsal **238** first tergite ventral **239** head and mesosoma dorsal **240** propodeum dorsal **241** head anterior **242** head and mesosoma lateral **243** apical hook of antenna lateral.

##### Distribution.

C and S Europe, but unknown from Sardinia (Giordani Soika and Borsato 1995); outside Europe known from Morocco, Israel, Lebanon, and Asia Minor. In Switzerland found up to 1920 m altitude (Neumeyer 2019) as in Greece (Arens 2012).

#### ﻿ Eumenes tripunctatus

Taxon classificationAnimaliaHymenopteraVespidae

(Christ, 1791)

8A1100D9-0192-50BB-8308-94E3E89AD44E

Figs 244–252 , 253–261

Sphex tripunctatus Christ, 1791: 317 (type series lost).
Eumenes (Eumenes) tripunctatus ; van der Vecht and Fischer 1972: 134; Gusenleitner 1972: 112–113; Fateryga 2017: 182, 2018: 209; Fateryga et al. 2020: 96–97.
Eumenes tripunctatus ; Tobias and Kurzenko 1978: 160; Fateryga 2010: 78.
Vespa trimaculata Lichtenstein, 1796: 202; van der Vecht and Fischer 1972: 134 (as synonym of E.tripunctatus; literature before 1972).
Eumenes venusta Fischer-Waldheim, 1843: 1, pl. 122; van der Vecht and Fischer 1972: 134 (as synonym of E.tripunctatus).

##### Note.

Conspicuous orange species only recently known to occur in Europe (Fateryga 2010, 2017, 2018; Fateryga et al. 2020).

##### Distribution.

Central Asia, European Russia, Ukraine (Crimea).

**Figures 244–252.**
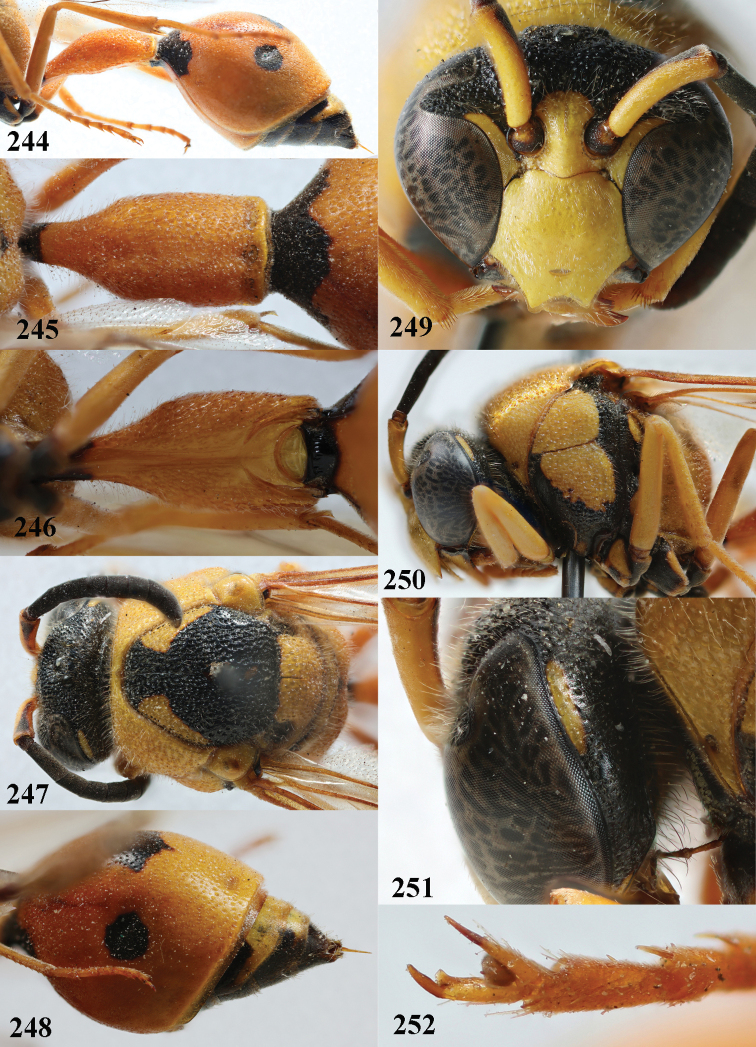
*Eumenestripunctatus* (Christ), Kazakhstan, female **244** metasoma lateral **245** first metasomal tergite dorsal **246** first tergite ventral **247** head and mesosoma dorsal **248** second metasomal tergite dorso-lateral **249** head anterior **250** head and mesosoma lateral **251** detail head and propleuron lateral **252** hind tarsal claw.

**Figures 253–261.**
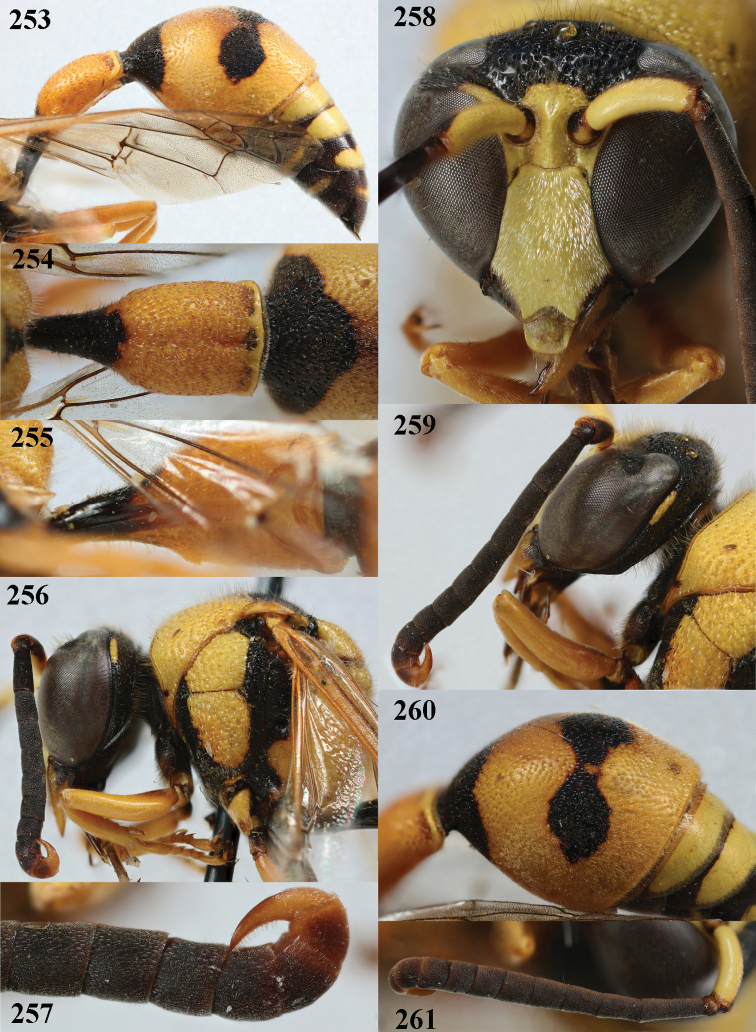
*Eumenestripunctatus* (Christ), Kazakhstan, male **253** metasoma lateral **254** first metasomal tergite dorsal **255** first tergite ventral **256** head and mesosoma lateral **257** apical hook of antenna lateral **258** head anterior **259** head and propleuron lateral. **260** second metasomal tergite dorso-lateral **261** antenna.

**Figures 262–266.**
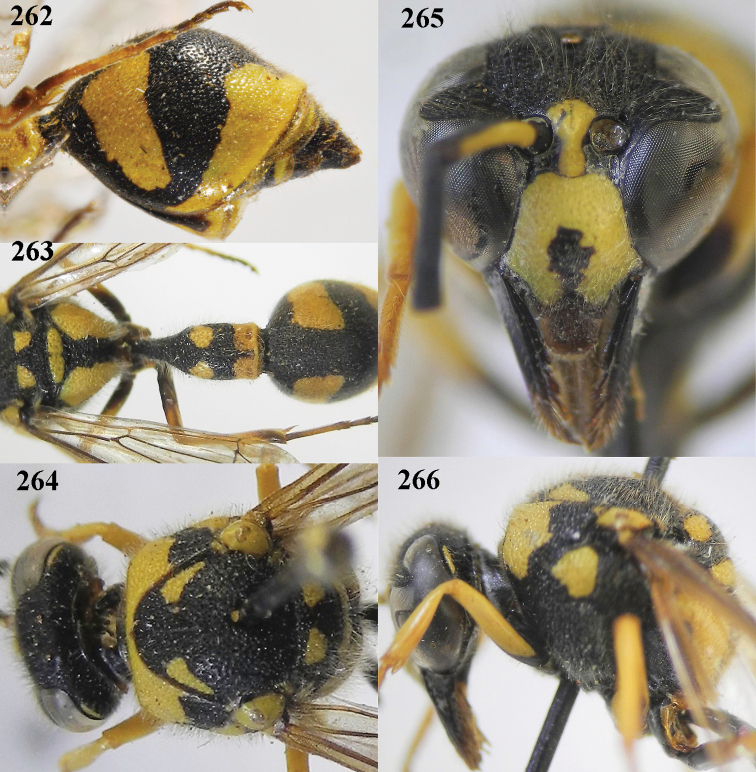
*Eumenesmediterraneusaemilianus* Guiglia, holotype, Italy, female **262** metasoma lateral **263** propodeum and first metasomal tergite dorsal **264** head and mesosoma dorsal **265** head anterior **266** head and mesosoma lateral. Photographs: R. Poggi (MSNG).

## Supplementary Material

XML Treatment for Eumenes c. coarctatus

XML Treatment for Eumenes coarctatus lunulatus

XML Treatment for Eumenes coronatus

XML Treatment for Eumenes cyrenaicus

XML Treatment for Eumenes dubius

XML Treatment for Eumenes mediterraneus

XML Treatment for Eumenes papillarius

XML Treatment for Eumenes pedunculatus

XML Treatment for Eumenes pomiformis

XML Treatment for Eumenes punctaticlypeus

XML Treatment for Eumenes sardous

XML Treatment for Eumenes sareptanus

XML Treatment for Eumenes subpomiformis

XML Treatment for Eumenes tripunctatus
